# Supplementary material for: B cell receptor and Toll-like receptor signaling coordinate to control distinct B-1 responses to both self and the microbiota
Source: eLife. 2019 Aug 21;8:e47015. doi: 10.7554/eLife.47015 (PMC6703855; doi:10.7554/eLife.47015)
Supplement: Figure 2—source data 1. — The variable heavy chain gene (VH gene), joining heavy chain gene (JH), heavy chain CDR3 peptide sequence, variable kappa chain gene (VH gene), joining kappa chain gene (JH gene), and kappa chain CDR3 peptide sequence for monoclonal antibodies generated from single-cell sorted Tomato– splenic B-1a cells from 6 wk old Ighg3T2A-Cre:TdTomato mice (n = 38). [file elife-47015-fig2-data1.docx]

| **Cell Identity** | **VH Gene** | **JH Gene** | **IgH CDR3 (AA)** | **VK Gene** | **JK gene** | **IgK CDR3 (AA)** |
| --- | --- | --- | --- | --- | --- | --- |
| Tom- B1a | IGHV1-53*01 | IGHJ2*01 | CAVSLQGYW | IGKV3-2*01 | IGKJ1*01 | CQQSKEVPWTF |
| Tom- B1a | IGHV1-54*01 | IGHJ2*01 | CARGDYYGSSYDYW | IGKV4-57*01 | IGKJ5*01 | CQQRSSYPLTF |
| Tom- B1a | IGHV1-36*01 | IGHJ2*01 | CARLQAQEVYFDYW | IGKV17-127*01 | IGKJ5*01 | CLQSDNMPLTF |
| Tom- B1a | IGHV1-7*01 | IGHJ2*03 | CARCPLILSMDYW | IGKV19-93*01 | IGKJ1*01 | CLQYDNLLWTF |
| Tom- B1a | IGHV6-6*01 | IGHJ3*01 | CTRRDSWFAYW | IGKV12-44*01 | IGKJ4*01 | CQHHYGTPFTF |
| Tom- B1a | IGHV11-2*01 | IGHJ1*03 | CMRYSNYWYFDVW | IGKV14-126*01 | IGKJ4*01 | CLQHGESPFTF |
| Tom- B1a | IGHV1-76*01 | IGHJ4*01 | CAGDAMDYW | IGKV3-2*01 | IGKJ1*01 | CQQSKEVPWTF |
| Tom- B1a | IGHV10-1*01 | IGHJ4*01 | CVRRNYYIPGLGHYYAMDYW | IGKV8-19*01 | IGKJ5*01 | CQNDYSYPLTF |
| Tom- B1a | IGHV11-2*01 | IGHJ1*03 | CMRYSNYWYFDVW | IGKV14-126*01 | IGKJ2*01 | CLQHGESPYTF |
| Tom- B1a | IGHV11-2*01 | IGHJ1*03 | CMRYSNYWYFDVW | IGKV14-126*01 | IGKJ2*01 | CLQHGESPYTF |
| Tom- B1a | IGHV6-6*01 | IGHJ3*01 | CTRGRIYYGNSPFAYW | IGKV8-27*01 | IGKJ1*01 | CHQYLSSWTF |
| Tom- B1a | IGHV6-3*01 | IGHJ2*03 | CTEGTIFDYW | IGKV12-44*01 | IGKJ2*01 | CQHHYGTPYTF |
| Tom- B1a | IGHV11-2*01 | IGHJ1*03 | CMRYSNYWYFDVW | IGKV14-126*01 | IGKJ2*01 | CLQHGESPYTF |
| Tom- B1a | IGHV1-5*01 | IGHJ1*03 | CTRGDYYGSSYVYFDVW | IGKV14-111*01 | IGKJ5*01 | CLQYDEFPLTF |
| Tom- B1a | IGHV10-1*01 | IGHJ2*01 | CVREPDYYGSSPDYW | IGKV9-120*01 | IGKJ5*01 | CLQYASSPLTF |
| Tom- B1a | IGHV14-2*01 | IGHJ2*01 | CALGNFDYW | IGKV8-30*01 | IGKJ2*01 | CQQYYSYYTF |
| Tom- B1a | IGHV1-64*01 | IGHJ2*01 | CASGSYYYGSSHYFDYX | IGKV4-68*01 | IGKJ2*01 | CQQWSSNPYTF |
| Tom- B1a | IGHV1-74*01 | IGHJ4*01 | CAIIYYSNYDAMDYW | IGKV4-91*01 | IGKJ5*01 | CQQGSSIPLTF |
| Tom- B1a | IGHV6-3*01 | IGHJ3*02 | CTGEGAGGWLRFW | IGKV12-44*01 | IGKJ4*01 | CQHHYGTPFTF |
| Tom- B1a | IGHV11-2*01 | IGHJ1*03 | CMRYSNYWYFDVW | IGKV14-126*01 | IGKJ4*01 | CLQHGESPFTF |
| Tom- B1a | IGHV11-2*01 | IGHJ1*03 | CMRYSSYWYFDVW | IGKV14-126*01 | IGKJ2*01 | CLQHGESPYTF |
| Tom- B1a | IGHV5-17*01 | IGHJ3*01 | CARNGYYAWFAYW | IGKV12-46*01 | IGKJ1*01 | CQHFWGTPWTF |
| Tom- B1a | IGHV10-1*01 | IGHJ1*01 | CVRLGWSYWYFDVW | IGKV3-2*01 | IGKJ2*01 | CQQSKEVPYTF |
| Tom- B1a | IGHV10-1*01 | IGHJ4*01 | CVRHAYGSTYYAMDYW | IGKV8-18*01 | IGKJ2*01 | CQHNHGSFLPYTF |
| Tom- B1a | IGHV1-55*01 | IGHJ4*01 | CANYDGYSHYYAMDYW | IGKV10-94*01 | IGKJ1*01 | CQQYSKLPRTF |
| Tom- B1a | IGHV6-3*01 | IGHJ4*01 | CTEDYGYAMDYW | IGKV12-46*01 | IGKJ1*01 | CQHFWGTPRTF |
| Tom- B1a | IGHV1-53*01 | IGHJ3*01 | CARGVGRWFAYW | IGKV3-2*01 | IGKJ1*01 | CQQXXEVPWTF |
| Tom- B1a | IGHV1-22*01 | IGHJ3*01 | CASPYSNYVWFAYW | IGKV3-5*01 | IGKJ1*01 | CQQSNEDPWTF |
| Tom- B1a | IGHV1-82*01 | IGHJ1*01 | CANGDRHYWYFDVW | IGKV3-2*01 | IGKJ2*01 | CQQSKEVPYTF |
| Tom- B1a | IGHV6-6*01 | IGHJ4*01 | CTRRYGSYYYAMDYW | IGKV12-46*01 | IGKJ2*01 | CQHFWGTPYTF |
| Tom- B1a | IGHV6-6*01 | IGHJ2*01 | CWSLHYW | IGKV12-46*01 | IGKJ2*01 | CQHFWGTPYTF |
| Tom- B1a | IGHV1-54*01 | IGHJ1*01 | CARRVVARYFDVW | IGKV4-57-1*01 | IGKJ5*01 | CQQYSGYPLTF |
| Tom- B1a | IGHV10-1*01 | IGHJ2*01 | CVGGGSSHYFDYW | IGKV3-5*01 | IGKJ2*01 | CQQSNEDPYTF |
| Tom- B1a | IGHV6-3*01 | IGHJ4*01 | CTEDYGYAMDYW | IGKV12-46*01 | IGKJ1*01 | CQHFWGTPRTF |
| Tom- B1a | IGHV1-75*01 | IGHJ2*03 | CARGAYW | IGKV8-19*01 | IGKJ5*01 | CQNDYSYPLTF |
| Tom- B1a | IGHV1-42*01 | IGHJ3*01 | CARWDYGSSSFAYW | IGKV14-126*01 | IGKJ4*01 | CLQHGESPFTF |
| Tom- B1a | IGHV6-6*01 | IGHJ3*01 | CTMAPFAYW | IGKV3-2*01 | IGKJ2*01 | CQQSKEVPYTF |
| Tom- B1a | IGHV3-6*01 | IGHJ3*01 | CAREGSLPWFAYW | IGKV4-59*01 | IGKJ1*01 | CQQWSSNPWTF |
