# Supplementary material for: B cell receptor and Toll-like receptor signaling coordinate to control distinct B-1 responses to both self and the microbiota
Source: eLife. 2019 Aug 21;8:e47015. doi: 10.7554/eLife.47015 (PMC6703855; doi:10.7554/eLife.47015)
Supplement: Figure 2—source data 2. — The variable heavy chain gene (VH gene), joining heavy chain gene (JH), heavy chain CDR3 peptide sequence, variable kappa chain gene (VH gene), joining kappa chain gene (JH gene), and kappa chain CDR3 peptide sequence for monoclonal antibodies generated from single-cell sorted Tomato+ splenic B-1a cells from 6 wk old Ighg3T2A-Cre:TdTomato mice (n = 48). [file elife-47015-fig2-data2.docx]

| **Cell Identity** | **VH gene** | **JH gene** | **IgH CDR3 (AA)** | **VK gene** | **JK gene** | **IgK CDR3 (AA)** |
| --- | --- | --- | --- | --- | --- | --- |
| Tom+ B1a | IGHV11-2*01 | IGHJ1*03 | CMRYGSSYWYFDVW | IGKV14-126*01 | IGKJ4*01 | CLQHGESPFTF |
| Tom+ B1a | IGHV6-6*0 | IGHJ2*01 | CTRNYGDYW | IGKV10-94*01 | IGKJ4*01 | CQQYSKLPFTF |
| Tom+ B1a | IGHV6-6*01 | IGHJ3*01 | CTRRDSWFAYW | IGKV12-44*01 | IGKJ4*01 | CQHHYGTPFTF |
| Tom+ B1a | IGHV2-9-1*01 | IGHJ1*03 | CARTYGSGYFDVW | IGKV12-41*01 | IGKJ2*01 | CQHFWSTPYTF |
| Tom+ B1a | IGHV11-2*01 | IGHJ1*01 | CMRYSNYWYFDVW | IGKV14-126*01 | IGKJ4*01 | CLQHGESPFTF |
| Tom+ B1a | IGHV6-3*01 | IGHJ3*01 | CTGQFAYW | IGKV6-25*01 | IGKJ1*01 | CQQHYSTPWTF |
| Tom+ B1a | IGHV3-8*01 | IGHJ1*03 | CARYGSGDRYFDVW | IGKV10-96*01 | IGKJ1*01 | CQQGNTF |
| Tom+ B1a | IGHV3-6*01 | IGHJ1*03 | CASDYYGYWYFDVW | IGKV15-103*01 | IGKJ4*01 | CQQGQSYPFTF |
| Tom+ B1a | IGHV1-15*01 | IGHJ1*03 | CTRSTGTFYWYFDVW | IGKV8-19*01 | IGKJ2*01 | CQNDYSYPYTF |
| Tom+ B1a | IGHV6-3*01 | IGHJ2*03 | CTGDLGRW | IGKV12-46*01 | IGKJ2*01 | CQHFWGTPYTF |
| Tom+ B1a | IGHV1-26*01 | IGHJ3*01 | CARDYNPWFAYW | IGKV4-92*01 | IGKJ2*01 | CQQGSSSPYTF |
| Tom+ B1a | IGHV11-2*01 | IGHJ1*03 | CMRYSSYWYFDVW | IGKV14-126*01 | IGKJ2*01 | CLQHGESPYTF |
| Tom+ B1a | IGHV6-6*01 | IGHJ3*01 | CTLPPWFAYW | IGKV12-44*01 | IGKJ2*01 | CQHHYGTPYTF |
| Tom+ B1a | IGHV11-2*01 | IGHJ1*03 | CMRYGGYWYFDVW | IGKV14-126*01 | IGKJ2*01 | CLQHGESPYTF |
| Tom+ B1a | IGHV11-2*01 | IGHJ1*03 | CMRYSNYWYFDVW | IGKV14-126*01 | IGKJ2*01 | CLQHGESPYTF |
| Tom+ B1a | IGHV11-2*01 | IGHJ1*01 | CMRYGNYWYFDXW | IGKV14-126*01 | IGKJ4*01 | CLQHGESPFTF |
| Tom+ B1a | IGHV3-6*01 | IGHJ4*01 | CASLLYGYAMDYW | IGKV4-80*01 | IGKJ1*01 | CHQWSSYPWTF |
| Tom+ B1a | IGHV1-26*01 | IGHJ2*01 | CARSDYYGSSSLDYW | IGKV4-91*01 | IGKJ5*01 | CQQGSSIPLTF |
| Tom+ B1a | IGHV1-15*01 | IGHJ1*01 | CTRSTGTFYWYFDVW | IGKV8-19*01 | IGKJ2*01 | CQNDYSYPYTF |
| Tom+ B1a | IGHV12-3*01 | IGHJ1*01 | CAGDYYGYWYFDVW | IGKV4-91*01 | IGKJ4*01 | CQQGSSIPFTF |
| Tom+ B1a | IGHV11-2*01 | IGHJ1*03 | CMRYSSYWYFDVW | IGKV15-103*01 | IGKJ1*01 | CQQGQSYPLTF |
| Tom+ B1a | IGHV3-1*01 | IGHJ2*01 | CARADFEYYYGSSYYFDYW | IGKV3-10*01 | IGKJ5*01 | CQQNNEDPLTF |
| Tom+ B1a | IGHV11-2*01 | IGHJ2*01 | CMRYDGYYYYFDYW | IGKV14-126*01 | IGKJ4*01 | CLQHGESPFTF |
| Tom+ B1a | IGHV11-2*01 | IGHJ1*03 | CMRYEGYHWYFDVW | IGKV14-126*01 | IGKJ2*01 | CLQHGESPYTF |
| Tom+ B1a | IGHV1-55*01 | IGHJ1*03 | CARYPSYYYGSSYWYFDVW | IGKV14-126*01 | IGKJ2*01 | CLQHGESPYTF |
| Tom+ B1a | IGHV6-3*01 | IGHJ2*03 | CTSLGLRGFDYW | IGKV12-44*01 | IGKJ1*01 | CQHHYGTPWTF |
| Tom+ B1a | IGHV6-6*01 | IGHJ2*03 | CWSLHYW | IGKV12-46*01 | IGKJ2*01 | CQHFWGTPYTF |
| Tom+ B1a | IGHV1-64*01 | IGHJ2*01 | CARSRGPYYFDYW | IGKV4-55*01 | IGKJ5*01 | CQQWSSYPLTF |
| Tom+ B1a | IGHV11-2*01 | IGHJ1*03 | CMRYSSYWYFDVW | IGKV14-126*01 | IGKJ2*01 | CLQHGESPYTF |
| Tom+ B1a | IGHV11-2*01 | IGHJ1*01 | CMRYSNYWYFDVW | IGKV14-126*01 | IGKJ4*01 | CLQHGESPFTF |
| Tom+ B1a | IGHV11-2*01 | IGHJ1*01 | CMRYSNYWYFDVW | IGKV14-126*01 | IGKJ2*01 | CLQHGESPYTF |
| Tom+ B1a | IGHV1-62-2*01 | IGHJ1*03 | CARHEDPYYYGSSPYWYFDVW | IGKV5-39*01 | IGKJ5*01 | CQNGHSFPLTF |
| Tom+ B1a | IGHV10-1*01 | IGHJ2*01 | CVRHSWESFDYW | IGKV6-23*01 | IGKJ1*01 | CQQYSSYPWTF |
| Tom+ B1a | IGHV3-6*01 | IGHJ4*01 | CARGVYAMDYW | IGKV10-96*01 | IGKJ1*01 | CQQGNTLPRTF |
| Tom+ B1a | IGHV1-81*01 | IGHJ4*01 | CAREGVTTVVATDYAMDYW | IGKV12-46*01 | IGKJ1*01 | CQHFWGTPRTF |
| Tom+ B1a | IGHV6-3*01 | IGHJ3*01 | CTVGGQGAWFAYW | IGKV12-44*01 | IGKJ5*01 | CQHHYGTPPTF |
| Tom+ B1a | IGHV10-3*01 | IGHJ4*01 | CVSGYAMDYW | IGKV3-10*01 | IGKJ5*01 | CQQNNEDPLTF |
| Tom+ B1a | IGHV6-6*01 | IGHJ3*01 | CTRREYPAWFAYW | IGKV8-30*01 | IGKJ1*01 | CQQYYSYPWTF |
| Tom+ B1a | IGHV2-3*01 | IGHJ2*03 | CAKLNGNPWYFDYW | IGKV10-96*01 | IGKJ1*01 | CQQGNTLPRTF |
| Tom+ B1a | IGHV1-76*01 | IGHJ2*03 | CARENPNFDYW | IGKV14-111*01 | IGKJ1*01 | CLQYDEFPRTF |
| Tom+ B1a | IGHV1-18*03 | IGHJ2*01 | CARRPPLYYYGSRKGYFDYW | IGKV8-19*01 | IGKJ4*01 | CQNDYSYPFTF |
| Tom+ B1a | IGHV6-3*01 | IGHJ2*01 | CASYGSSSWYFDVW | IGKV12-44*01 | IGKJ1*01 | CQHHYGTPWTF |
| Tom+ B1a | IGHV1-19*01 | IGHJ4*01 | CARSMITRAMDYW | IGKV5-48*01 | IGKJ5*01 | CQQSNSWPLTF |
| Tom+ B1a | IGHV10-1*01 | IGHJ2*03 | CVRQGDYYGSSRYFDYW | IGKV17-121*01 | IGKJ2*01 | CLQSDNLPYTF |
| Tom+ B1a | IGHV6-6*01 | IGHJ1*01 | CARAHQVAAAGTWTFDYW | IGKV12-44*01 | IGKJ1*01 | CQHHYGTPWTF |
| Tom+ B1a | IGHV6-6*01 | IGHJ2*01 | CTGGYPFAYW | IGKV12-44*01 | IGKJ2*01 | CQHHYGTPYTF |
| Tom+ B1a | IGHV2-2*01 | IGHJ4*01 | CARNDYDVLFYYAMDYW | IGKV8-19*01 | IGKJ5*01 | CQNDYSYPLTF |
| Tom+ B1a | IGHV6-3*01 | IGHJ3*01 | CTGPYGGFAYW | IGKV5-43*01 | IGKJ5*01 | CQQSNSWPLTF |
