# Supplementary material for: B cell receptor and Toll-like receptor signaling coordinate to control distinct B-1 responses to both self and the microbiota
Source: eLife. 2019 Aug 21;8:e47015. doi: 10.7554/eLife.47015 (PMC6703855; doi:10.7554/eLife.47015)
Supplement: Figure 6—source data 1. — The CDR3 peptide sequence, variable heavy chain gene, joining heavy chain gene, total read counts (copy), normalized read counts (norm.copy), and the sum of normalized counts of PtC-binding CDR3 peptide sequences in peritoneal cavity (PerC) B-1a samples from 7 wk old WT (black), Tlr2-/-Tlr4-/-Unc93b13d/3d (blue), Tlr2-/-Tlr4-/- (pink), and Unc93b13d/3d (green) mice. PtC-binding CDR3 peptide sequences include MRYSNYWYFDV (highlighted in blue), MRYGSSYWYFDV (highlighted in orange), and MRYGNYWYFDV (highlighted in purple). CDR3 sequences in italic font denotes that they did not appear in the top 10 CDR3 sequences for that sample, but were included to be able to determine the sum of PtC-binding CDR3 sequencing reads. Sequencing reads were normalized by artificially scaling to 10 million reads to account for differences in read depth among samples. There are three biological replicates for each genotype in one experiment. [file elife-47015-fig6-data1.docx]

# Sum PtC- binding

**Sum PtC- binding**

**Sum PtC- binding**

**PerC WT 1 Copy Norm. Copy CDR3 PerC WT 2 Copy Norm. Copy CDR3 PerC WT 3 Copy Norm. Copy CDR3**

ARYYYGSSYAMDY mIGHV7-3 mIGHJ4 38622 599824.81 **790,370** MRYSNYWYFDV mIGHV11-2 mIGHJ1 32547 550678.98 **1,055,675** MRYSNYWYFDV mIGHV11-2 mIGHJ1 47370 675768.21 **1,446,461**

MRYSNYWYFDV mIGHV11-2 mIGHJ1 29398 456570.09 VYGNWYFDV mIGHV9-1 mIGHJ1 29329 496232.03 MRYGNYWYFDV mIGHV11-2 mIGHJ1 45491 648962.88

ARRDYGSSYWYFDV mIGHV1-55 mIGHJ1 23938 371772.73 MRYGNYWYFDV mIGHV11-2 mIGHJ1 28898 488939.72 ARRYYGSSYWYFDV mIGHV1-55 mIGHJ1 18979 270749.52

MRYGNYWYFDV mIGHV11-2 mIGHJ1 13808 214447.23 ARFYYYGSSYAMDY mIGHV1-55 mIGHJ4 22792 385629.25 ARIYYGNYWYFDV mIGHV1-55 mIGHJ1 17559 250492.17

ARHYYGSSYYFDY mIGHV5-6-1 mIGHJ2 8432 130954.45 VRGDGTIDY mIGHV10-1 mIGHJ2 14121 238920.27 MRYGSSYWYFDV mIGHV11-2 mIGHJ1 8533 121729.58

MRYGSSYWYFDV mIGHV11-2 mIGHJ1 7685 119353.06 ASGNYVY mIGHV1-53 mIGHJ2 12891 218109.28 ARCYYGSSYWYFDV mIGHV1-55 mIGHJ1 6073 86635.853

ARNYYYMDY mIGHV2-2 mIGHJ4 5174 80355.59 ARHYYGSSYYFDY mIGHV5-6-1 mIGHJ2 11861 200682.19 ARYYYGSSYAMDY mIGHV7-3 mIGHJ4 5614 80087.877

AKRGNYYAMDY mIGHV2-9 mIGHJ4 5051 78445.32 VRDGFAY mIGHV10-3 mIGHJ3 7984 135085.29 ARMGYHAMDY mIGHV7-3 mIGHJ4 5439 77591.372

MRYPYSNYWYFDV mIGHV11-2 mIGHJ1 5010 77808.563 ASYAMDY mIGHV7-3 mIGHJ4 7229 122311.07 ARRDYGSSYWYFDV mIGHV1-55 mIGHJ1 5410 77177.666

AIYYDYYYAMDY mIGHV1-4 mIGHJ4 4938 76690.356 ARYYYGSSYAMDY mIGHV7-3 mIGHJ4 6423 108673.95 VRGDGTLDY mIGHV10-1 mIGHJ2 5375 76678.365

*MRYGSSYWYFDV mIGHV11-2 mIGHJ1 949* 16056.606

# Sum PtC- binding

**Sum PtC- binding**

**Sum PtC- binding**

**PerC 2x4dKO 1 Copy Norm. Copy CDR3 PerC 2x4dKO 2 Copy Norm. Copy CDR3 PerC 2x4dKO 2 Copy Norm. Copy CDR3**

MRYSNYWYFDV mIGHV11-2 mIGHJ1 90492 1361048 **1,928,588** ARIYYGSSYWYFDV mIGHV1-55 mIGHJ1 138504 2008867.8 **1,654,752** ARRSNWYFDV mIGHV7-3 mIGHJ1 143982 2215772.4 **912,966**

ARYYYGSSYAMDY mIGHV7-3 mIGHJ4 27036 406635.88 MRYSNYWYFDV mIGHV11-2 mIGHJ1 73579 1067192.9 MRYGNYWYFDV mIGHV11-2 mIGHJ1 34278 527512.1

MRYGSSYWYFDV mIGHV11-2 mIGHJ1 21260 319761.76 MRYGNYWYFDV mIGHV11-2 mIGHJ1 28896 419108.78 MRYDGYYWYFDV mIGHV11-2 mIGHJ1 19542 300736.37

MRYGNYWYFDV mIGHV11-2 mIGHJ1 16474 247777.76 ARYYYGSSYAMDY mIGHV7-3 mIGHJ4 17683 256474.97 MRYSNYWYFDV mIGHV11-2 mIGHJ1 15503 238579.27

VTGDY mIGHV10-3 mIGHJ2 10364 155880.1 ARRYYGSSYWYFDV mIGHV1-55 mIGHJ1 13521 196109.15 ARYNWDAMDY mIGHV7-3 mIGHJ4 11805 181669.89

AKLGNYAMDY mIGHV2-9 mIGHJ4 9451 142148.09 MRYGSSYWYFDV mIGHV11-2 mIGHJ1 11614 168449.94 ARRDYGSSYWYFDV mIGHV1-55 mIGHJ1 11076 170451.14

ASGRFAY mIGHV1-53 mIGHJ3 8664 130311.19 AKHYYGSYYAMDY mIGHV2-9 mIGHJ4 10925 158456.65 ARYYYGSSYAMDY mIGHV7-3 mIGHJ4 10374 159647.89

ARYNWDAMDY mIGHV7-3 mIGHJ4 7744 116473.9 ARFYYYGSSYAMDY mIGHV1-55 mIGHJ4 6236 90447.203 MRYGSSYWYFDV mIGHV11-2 mIGHJ1 9544 146874.83

ARWANYWYFDV mIGHV7-3 mIGHJ1 6868 103298.39 ASGNFAY mIGHV1-19 mIGHJ3 5307 76972.949 MRYGPYWYFDV mIGHV11-2 mIGHJ1 8615 132578.23

ARYYYGSSWYFDV mIGHV1-76 mIGHJ1 6800 102275.63 ARSPYDYFDY mIGHV7-3 mIGHJ2 5045 73172.89 ARNWDY mIGHV1-66 mIGHJ2 6867 105677.86

# Sum PtC- binding

**Sum PtC- binding**

**Sum PtC- binding**

**PerC Unc93B1 1 Copy Norm. Copy CDR3 PerC Unc93B1 2 Copy Norm. Copy CDR3 PerC Unc93B1 2 Copy Norm. Copy CDR3**

ARYNWDAMDY mIGHV7-3 mIGHJ4 26730 416024.65 **698,090** ARDHWGFDY mIGHV1-4 mIGHJ2 72545 1019825.8 **163,029** ARFYYYGSSYAMDY mIGHV1-55 mIGHJ4 115273 1691651.1 **207,008**

MRYGNYWYFDV mIGHV11-2 mIGHJ1 20290 315792.75 ARYYYGSSYAMDY mIGHV7-3 mIGHJ4 45599 641023.3 ARYYYGSSYAMDY mIGHV7-3 mIGHJ4 39114 574004.69

MRYSNYWYFDV mIGHV11-2 mIGHJ1 19215 299061.49 ARSYGSSYWYFDV mIGHV1-84 mIGHJ1 31182 438351.47 ARIVYGNPYYFDY mIGHV8-8 mIGHJ2 37276 547031.73

ARYYYGSSYAMDY mIGHV7-3 mIGHJ4 12384 192744.08 ARRIYYGNGHAMDY mIGHV1-55 mIGHJ4 23116 324960.95 ARVDWDYFDY mIGHV3-6 mIGHJ2 14911 218821.5

TVYYSNSYFDY mIGHV6-3 mIGHJ2 12354 192277.16 AGTFIDY mIGHV1-77 mIGHJ2 22350 314192.65 ASYYDYYYAMDY mIGHV1-4 mIGHJ4 12206 179125.15

AGDYDGYWYFDV mIGHV12-3 mIGHJ1 9678 150628.01 ARDYSDY mIGHV1-7 mIGHJ2 22310 313630.34 MRYGNYWYFDV mIGHV11-2 mIGHJ1 10402 152651.14

ARDYSNYWYFDV mIGHV1-64 mIGHJ1 9055 140931.66 ARGGFIG mIGHV1-85 mIGHJ4 21238 298560.34 ARSQSYYFDY mIGHV9-3 mIGHJ2 9312 136655.21

ARYYYGSSWYFDV mIGHV1-76 mIGHJ1 6741 104916.65 ARFYYYGSNYAMDY mIGHV1-55 mIGHJ4 14639 205792.67 ARDSSGYYFDY mIGHV1-36 mIGHJ2 9139 134116.4

AGDRLGYWYFDV mIGHV12-3 mIGHJ1 6505 101243.56 ARYKGITTRYFDV mIGHV7-3 mIGHJ1 9254 130091.22 VANFDY mIGHV10-1 mIGHJ2 6987 102535.43

ARRYYYGSSYYFDY mIGHV1-55 mIGHJ2 6057 94270.906 MRYSNYWYFDV mIGHV11-2 mIGHJ1 9059 127349.94 ARSKDYDYFDY mIGHV1-64 mIGHJ2 5904 86642.218

*MRYSNYWYFDV mIGHV11-2 mIGHJ1*

*MRYGSSYWYFDV mIGHV11-2 mIGHJ1*

*2955 43365.134*

*749 10991.704*

*MRYGSSYWYFDV mIGHV11-2 mIGHJ1 5348 83236.059*

*MRYGNYWYFDV mIGHV11-2 mIGHJ1 1294 18190.841*

*MRYGSSYWYFDV mIGHV11-2 mIGHJ1 1244 17487.949*

# Sum PtC- binding

**Sum PtC- binding**

**Sum PtC- binding**

**PerC TLR KO 1 Copy Norm. Copy CDR3 PerC TLR KO 2 Copy Norm. Copy CDR3 PerC TLR KO 3 Copy Norm. Copy CDR3**

ARYYYGSSYAMDY mIGHV7-3 mIGHJ4 25929 415329.17 **375,044** ARWGGSAMDY mIGHV5-15 mIGHJ4 57013 1222041.5 **625,457** ARRNYGSSYWYFDV mIGHV1-55 mIGHJ1 34668 654869.4 **345,852**

AREDYYSNYCFDY mIGHV5-16 mIGHJ2 25529 408921.99 AKLNNYYAMDY mIGHV2-9 mIGHJ4 27039 579565.7 AKRGAQATFAY mIGHV2-9 mIGHJ3 27130 512478.56

ARRDYGSSYWYFDV mIGHV1-55 mIGHJ1 16240 260131.35 MRYSNYWYFDV mIGHV11-2 mIGHJ1 13764 295023.57 ARYYYGSSYAMDY mIGHV7-3 mIGHJ4 23808 449726.85

TRFPY mIGHV6-6 mIGHJ2 14529 232724.65 ARKDYYGSSYYFDY mIGHV1-55 mIGHJ2 12080 258927.98 VSGSSYFDY mIGHV10-1 mIGHJ2 10895 205803.68

MRYSNYWYFDV mIGHV11-2 mIGHJ1 13027 208665.71 VRQNWDPYYFDY mIGHV10-1 mIGHJ2 11714 251082.97 ARRAYYGSSYYFDY mIGHV1-55 mIGHJ2 10455 197492.2

ARFYYYGSSYAMDY mIGHV1-55 mIGHJ4 11885 190373.22 MRYGSSYWYFDV mIGHV11-2 mIGHJ1 10170 217988.21 MRYGNYWYFDV mIGHV11-2 mIGHJ1 8992 169856.51

MRYDGYYWYFDV mIGHV11-2 mIGHJ1 10196 163318.92 ARGDDFYFDY mIGHV1-36 mIGHJ2 9885 211879.39 MRYSNYWYFDV mIGHV11-2 mIGHJ1 8584 162149.5

TTFIPY mIGHV6-6 mIGHJ2 9706 155470.13 ARFYYYGSSYAMDY mIGHV1-55 mIGHJ4 8580 183907.45 AKQGDYWYFDV mIGHV2-9 mIGHJ1 7428 140312.97

ARYYDYDYAMDY mIGHV1-4 mIGHJ4 8159 130690.37 ARHAGSAMDY mIGHV5-15 mIGHJ4 6120 131178.74 AIYYDYDYAMDY mIGHV1-4 mIGHJ4 7074 133626

MRYGNYWYFDV mIGHV11-2 mIGHJ1 7906 126637.83 ARSGSYYFDY mIGHV1-82 mIGHJ2 6093 130600.01 AIQGY mIGHV1-74 mIGHJ2 6788 128223.53

*MRYGNYWYFDV mIGHV11-2 mIGHJ1 5246 112445.05*

*MRYGSSYWYFDV mIGHV11-2 mIGHJ1 733 13846.177*

*MRYGSSYWYFDV mIGHV11-2 mIGHJ1 2481 39740.509*
