# Supplementary material for: B cell receptor and Toll-like receptor signaling coordinate to control distinct B-1 responses to both self and the microbiota
Source: eLife. 2019 Aug 21;8:e47015. doi: 10.7554/eLife.47015 (PMC6703855; doi:10.7554/eLife.47015)
Supplement: Figure 6—source data 2. — The CDR3 peptide sequence, variable heavy chain gene, joining heavy chain gene, total read counts (copy), normalized read counts (norm.copy), and the sum of normalized counts of PtC-binding CDR3 peptide sequences in spleen (Spl) B-1a samples from 7 wk old WT (black), Tlr2-/-Tlr4-/-Unc93b13d/3d (blue), Tlr2-/-Tlr4-/- (pink), and Unc93b13d/3d (green) mice. PtC-binding CDR3 peptide sequences include MRYSNYWYFDV (highlighted in blue), MRYGSSYWYFDV (highlighted in orange), and MRYGNYWYFDV (highlighted in purple). CDR3 sequences in italic font denotes that they did not appear in the top 10 CDR3 sequences for that sample, but were included to be able to determine the sum of PtC-binding CDR3 sequencing reads. Sequencing reads were normalized by artificially scaling to 10 million reads to account for differences in read depth among samples. There are three biological replicates for each genotype in one experiment. [file elife-47015-fig6-data2.docx]

# Sum PtC- binding

**Sum PtC- binding**

**Sum PtC- binding**

**Spleen WT 1 Copy Norm. Copy CDR3 Spleen WT 2 Copy Norm. Copy CDR3 Spleen WT 3 Copy Norm. Copy CDR3**

MRYSNYWYFDV mIGHV11-2 mIGHJ1 22839 432,331 **807,324** MRYSNYWYFDV mIGHV11-2 mIGHJ1 38858 693,750 **1,124,376** ARIYYGNYWYFDV mIGHV1-55 mIGHJ1 92058 1,505,229 **1,172,816**

MRYGSSYWYFDV mIGHV11-2 mIGHJ1 14534 275,121 MRYGNYWYFDV mIGHV11-2 mIGHJ1 15554 277,693 MRYSNYWYFDV mIGHV11-2 mIGHJ1 56235 919,492

ARYYYGSSYAMDY mIGHV7-3 mIGHJ4 7719 146,117 VSNYEY mIGHV10-1 mIGHJ3 8613 153,772 ARRYYGSSYWYFDV mIGHV1-55 mIGHJ1 55654 909,992

ARRDYGSSYWYFDV mIGHV1-55 mIGHJ1 6952 131,598 MRYGSSYWYFDV mIGHV11-2 mIGHJ1 8566 152,933 ARCYYGSSYWYFDV mIGHV1-55 mIGHJ1 11692 191,174

MRYPYSNYWYFDV mIGHV11-2 mIGHJ1 6084 115,167 VSNYDYAMDY mIGHV1-75 mIGHJ4 7822 139,650 MRYGNYWYFDV mIGHV11-2 mIGHJ1 9382 153,404

MRYGNYWYFDV mIGHV11-2 mIGHJ1 5276 99,872 MRYGEYWYFDV mIGHV11-2 mIGHJ1 5863 104,675 ARRDYGSSYWYFDV mIGHV1-55 mIGHJ1 8142 133,129

AKEGYSNYYAMDY mIGHV2-9 mIGHJ4 4864 92,073 VRGDGTIDY mIGHV10-1 mIGHJ2 5677 101,354 VRGDGTLDY mIGHV10-1 mIGHJ2 6857 112,118

ARNYYYMDY mIGHV2-2 mIGHJ4 4399 83,271 VRGDGTVDY mIGHV10-1 mIGHJ2 4742 84,661 TEGYLGPNWYFDV mIGHV6-3 mIGHJ1 6531 106,788

TGYYSNYYAMDY mIGHV6-3 mIGHJ4 3940 74,582 VYGNWYFDV mIGHV9-1 mIGHJ1 4672 83,411 MRYGSSYWYFDV mIGHV11-2 mIGHJ1 6111 99,920

ARRYYGSSYWYFDV mIGHV1-55 mIGHJ1 3407 64,493 ARRSNYVFDY mIGHV7-3 mIGHJ2 4229 75,502 ARGYYGSSLWYFDV mIGHV1-75 mIGHJ1 4942 80,806

# Sum PtC- binding

**Sum PtC- binding**

**Sum PtC- binding**

**Spleen 2x4dKO 1 Copy Norm. Copy CDR3 Spleen 2x4dKO 2 Copy Norm. Copy CDR3 Spleen 2x4dKO 3 Copy Norm. Copy CDR3**

MRYSNYWYFDV mIGHV11-2 mIGHJ1 63754 942,598 **1,357,640** ARRSNWYFDV mIGHV7-3 mIGHJ1 142668 2,206,451 **529,636** MRYSNYWYFDV mIGHV11-2 mIGHJ1 75387 1,139,289 **1,568,697**

MRYGSSYWYFDV mIGHV11-2 mIGHJ1 22471 332,232 MRYDGYYWYFDV mIGHV11-2 mIGHJ1 29154 450,885 ARSNWDGYFDV mIGHV7-3 mIGHJ1 39295 593,847

ARFITTVVAPYYFDY mIGHV7-3 mIGHJ2 11416 168,785 MRYGNYWYFDV mIGHV11-2 mIGHJ1 22187 343,136 ARIYYGSSYWYFDV mIGHV1-55 mIGHJ1 37667 569,244

MRYGSSWYFDV mIGHV11-2 mIGHJ1 8673 128,230 ARSITTVPFAY mIGHV1-55 mIGHJ3 18009 278,521 ARRYYGSSYWYFDV mIGHV1-55 mIGHJ1 25357 383,209

ARYRTAQALYYFDY mIGHV7-3 mIGHJ2 8526 126,056 SSYAMDY mIGHV7-3 mIGHJ4 10619 164,230 MRYGNYWYFDV mIGHV11-2 mIGHJ1 20309 306,921

VRHIGSSYFDY mIGHV10-1 mIGHJ2 7269 107,472 MRYSNYWYFDV mIGHV11-2 mIGHJ1 8300 128,365 ARDWDYWYFDV mIGHV1-7 mIGHJ1 16640 251,473

ARYYYGSSYAMDY mIGHV7-3 mIGHJ4 6383 94,372 ARSLYDGYYDY mIGHV7-3 mIGHJ2 5801 89,716 ARMSTVVATGYFDY mIGHV7-3 mIGHJ2 12828 193,864

ASYAMDY mIGHV7-3 mIGHJ4 6276 92,790 ARNYYSGFDY mIGHV7-3 mIGHJ2 5400 83,514 ASSPQGYFDY mIGHV7-3 mIGHJ2 9219 139,323

MRYGNYWYFDV mIGHV11-2 mIGHJ1 5601 82,810 ASYAMDY mIGHV7-3 mIGHJ4 5008 77,452 MRYGSSYWYFDV mIGHV11-2 mIGHJ1 8105 122,487

ARYNSNPYYFDY mIGHV7-3 mIGHJ2 4820 71,263 AKKGYAMDY mIGHV2-3 mIGHJ4 4949 76,539 ATLRSAMDY mIGHV2-9 mIGHJ4 7159 108,191

*MRYGSSYWYFDV mIGHV11-2 mIGHJ1 3759 58,135*

# Sum PtC- binding

**Sum PtC- binding**

**Sum PtC- binding**

**Spleen Unc93B1 1 Copy Norm. Copy CDR3 Spleen Unc93B1 2 Copy Norm. Copy CDR3 Spleen Unc93B1 3 Copy Norm. Copy CDR3**

ARYNWDAMDY mIGHV7-3 mIGHJ4 34075 530,183 **501,165** ARRIYYGNGHAMDY mIGHV1-55 mIGHJ4 73643 1,064,301 **92,436** ARPYYYGSSLDY mIGHV1-80 mIGHJ2 55399 790,421 **174,281**

MRYSNYWYFDV mIGHV11-2 mIGHJ1 12350 192,157 ARDHWGFDY mIGHV1-4 mIGHJ2 39618 572,566 AKHGYRGYFDV mIGHV2-9 mIGHJ1 21195 302,406

MRYGSSYWYFDV mIGHV11-2 mIGHJ1 10055 156,449 ARFYYGSSYAMDY mIGHV1-53 mIGHJ4 26278 379,774 ARYYYGSSYAMDY mIGHV7-3 mIGHJ4 14642 208,909

ARRDYYGSIYAMDY mIGHV1-55 mIGHJ4 9993 155,484 ARTGIAY mIGHV9-4 mIGHJ3 22914 331,157 ARDSSGYYFDY mIGHV1-36 mIGHJ2 14513 207,068

MRYGNYWYFDV mIGHV11-2 mIGHJ1 9805 152,559 AQLVY mIGHV1-55 mIGHJ2 13203 190,812 ARFYYYGSSYAMDY mIGHV1-55 mIGHJ4 11025 157,302

ASYSNFDY mIGHV3-6 mIGHJ2 8035 125,019 ARYYYGSSYAMDY mIGHV7-3 mIGHJ4 7478 108,073 MRYDGYYWYFDV mIGHV11-2 mIGHJ1 9529 135,958

ARYYYGSSYAMDY mIGHV7-3 mIGHJ4 5907 91,909 ARSYGSSYWYFDV mIGHV1-84 mIGHJ1 7382 106,686 ARTLYGPLFAY mIGHV1-55 mIGHJ3 8546 121,932

ARSTTVVATPFDY mIGHV1-42 mIGHJ2 5213 81,111 ARDDYDY mIGHV1-61 mIGHJ2 6840 98,853 MRYGNYWYFDV mIGHV11-2 mIGHJ1 7253 103,484

AITTVVSYWYFDV mIGHV1-53 mIGHJ1 3987 62,035 ARGGFIG mIGHV1-85 mIGHJ4 5865 84,762 AREWLLPFAY mIGHV1-72 mIGHJ3 7221 103,028

AKQGY mIGHV9-3 mIGHJ2 3854 59,965 MRYSNYWYFDV mIGHV11-2 mIGHJ1 4995 72,189 ARSQSYYFDY mIGHV9-3 mIGHJ2 6624 94,510

*MRYGSSYWYFDV mIGHV11-2 mIGHJ1 1147 16,577 MRYGSSYWYFDV mIGHV11-2 mIGHJ1 1554 22,172*

*MRYGNYWYFDV mIGHV11-2 mIGHJ1 254 3,671 MRYSNYWYFDV mIGHV11-2 mIGHJ1 3408 48,625*

# Sum PtC- binding

**Sum PtC- binding**

**Sum PtC- binding**

**Spleen TLR KO 1 Copy Norm. Copy CDR3 Spleen TLR KO 2 Copy Norm. Copy CDR3 Spleen TLR KO 3 Copy Norm. Copy CDR3**

ARYYYGSSYAMDY mIGHV7-3 mIGHJ4 299226 4,283,156 **180,487** ARKDYYGSSYYFDY mIGHV1-55 mIGHJ2 15376 400,171 **473,407** ARGYGSSLWYFDV mIGHV1-84 mIGHJ1 72211 1,221,774 **203,034**

ARRGNYDWYFDV mIGHV1-55 mIGHJ1 25412 363,750 ARGDDFYFDY mIGHV1-36 mIGHJ2 12852 334,482 ARRAYYGSSYYFDY mIGHV1-55 mIGHJ2 36565 618,662

AREDYYSNYCFDY mIGHV5-16 mIGHJ2 21992 314,796 MRYSNYWYFDV mIGHV11-2 mIGHJ1 11987 311,970 AIQGY mIGHV1-74 mIGHJ2 29345 496,503

MRYSNYWYFDV mIGHV11-2 mIGHJ1 7417 106,168 AKLNNYYAMDY mIGHV2-9 mIGHJ4 11036 287,219 TGEYRV mIGHV6-3 mIGHJ1 25894 438,114

MRYDGYYWYFDV mIGHV11-2 mIGHJ1 6629 94,888 TRWDTHWYFDV mIGHV1-5 mIGHJ1 10824 281,702 ARGGYYYGSMYAMD mIGHV1-55 mIGHJ4 14866 251,525

ARSKIYLDY mIGHV1-63 mIGHJ2 6210 88,891 ARWGGSAMDY mIGHV5-15 mIGHJ4 10150 264,161 ARVYYGSSYWYFDV mIGHV1-55 mIGHJ1 11755 198,889

ARGDY mIGHV1-58 mIGHJ2 5691 81,462 ARDLSYWYFDV mIGHV1-55 mIGHJ1 9990 259,996 AREGLPAFDY mIGHV1-75 mIGHJ2 9857 166,776

VSSYSYAMDY mIGHV10-1 mIGHJ4 4108 58,802 ARSGYYGSSLIPLYYY mIGHV1-53 mIGHJ4 8884 231,212 AREGWLLLTLFDY mIGHV3-6 mIGHJ2 9231 156,184

MRYGYGYWYFDV mIGHV11-2 mIGHJ1 3666 52,476 AKREDYYYGSRGAW mIGHV2-9 mIGHJ3 7336 190,924 ARSDGSSWYFDV mIGHV1-22 mIGHJ1 8488 143,613

ASRARNDYTWFAY mIGHV7-3 mIGHJ3 3407 48,768 ARSTMITNY mIGHV1-64 mIGHJ2 6455 167,996 MRYGNYWYFDV mIGHV11-2 mIGHJ1 6797 115,002

*MRYGSSYWYFDV mIGHV11-2 mIGHJ1*

*MRYGNYWYFDV mIGHV11-2 mIGHJ1*

*3159*

*2033*

*45,218*

*29,101*

*MRYGSSYWYFDV mIGHV11-2 mIGHJ1*

*MRYGNYWYFDV mIGHV11-2 mIGHJ1*

*4942*

*1261*

*128,619*

*32,818*

*MRYSNYWYFDV mIGHV11-2 mIGHJ1 4254 71,976*

*MRYGSSYWYFDV mIGHV11-2 mIGHJ1 949 16,057*
