# Supplementary material for: B cell receptor and Toll-like receptor signaling coordinate to control distinct B-1 responses to both self and the microbiota
Source: eLife. 2019 Aug 21;8:e47015. doi: 10.7554/eLife.47015 (PMC6703855; doi:10.7554/eLife.47015)
Supplement: Figure 6—source data 3. — Table summarizing the variable heavy chain gene usage frequency in peritoneal cavity and spleen B-1a samples from 7 wk old WT (black), Tlr2-/-Tlr4-/-Unc93b13d/3d (blue), Tlr2-/-Tlr4-/- (pink), and Unc93b13d/3d (green) mice. There are three biological replicates for each genotype in one experiment. [file elife-47015-fig6-data3.docx]

|  | **Peritoneal Cavity B-1a** | | | | | | | | | | | |
| --- | --- | --- | --- | --- | --- | --- | --- | --- | --- | --- | --- | --- |
| **VH Gene** | **WT 1** | **WT 2** | **WT 3** | **Unc93B1 1** | **Unc93B1 2** | **Unc93B1 3** | **TLR KO 1** | **TLR KO 2** | **TLR KO 3** | **2x4dKO 1** | **2x4dKO 2** | **2x4dKO 3** |
| mIGHV1-4 | 1.2 | 1.4 | 0.8 | 0.9 | 11.7 | 2.8 | 2 | 0.4 | 2 | 0.9 | 1 | 0.1 |
| mIGHV1-5 | 0.2 | 0.1 | 0.1 | 0.8 | 0.3 | 0.1 | 0.1 | 0.3 | 0.1 | 0.5 | 0.2 | 0 |
| mIGHV1S5 | 0 | 0 | 0 | 0 | 0 | 0 | 0 | 0 | 0 | 0 | 0 | 0 |
| mIGHV1-7 | 1.8 | 1.2 | 0.7 | 1.1 | 4.3 | 1.4 | 0.7 | 0.6 | 0.7 | 0.7 | 2 | 1 |
| mIGHV1-9 | 1.1 | 0.8 | 0.4 | 1.7 | 0.9 | 0.4 | 0.8 | 1.4 | 0.8 | 1.1 | 0.3 | 0.1 |
| mIGHV1-11 | 0.2 | 0.1 | 0.2 | 0.1 | 0 | 0.1 | 0.1 | 0 | 0.1 | 0.2 | 0 | 0 |
| mIGHV1-12 | 0.2 | 0.5 | 0.1 | 0.1 | 0.1 | 0.1 | 0 | 0.1 | 0 | 0.1 | 0 | 0.3 |
| mIGHV1S12 | 0 | 0 | 0 | 0 | 0 | 0 | 0 | 0 | 0 | 0 | 0 | 0 |
| mIGHV1-14 | 0 | 0 | 0 | 0 | 0 | 0 | 0 | 0 | 0 | 0 | 0 | 0 |
| mIGHV1S14 | 0 | 0 | 0 | 0 | 0 | 0 | 0 | 0 | 0 | 0 | 0 | 0 |
| mIGHV1-15 | 0 | 0 | 0 | 0 | 0 | 0 | 0 | 0 | 0 | 0 | 0 | 0 |
| mIGHV1-17-1 | 0 | 0 | 0 | 0 | 0 | 0 | 0 | 0 | 0 | 0 | 0 | 0 |
| mIGHV1-18 | 0.4 | 0.8 | 0.3 | 1.4 | 0.5 | 0.5 | 0.4 | 0.2 | 0.4 | 1 | 0.7 | 0.4 |
| mIGHV1-19 | 4.4 | 5.4 | 4.8 | 2.2 | 1.2 | 1.8 | 1.4 | 1.7 | 1.4 | 3.8 | 3.8 | 3.2 |
| mIGHV1-20 | 0.3 | 0 | 0.1 | 0.2 | 0.1 | 0.1 | 0 | 0.1 | 0 | 0.1 | 0.4 | 0.2 |
| mIGHV1S20 | 0 | 0 | 0 | 0 | 0 | 0 | 0 | 0 | 0 | 0 | 0 | 0 |
| mIGHV1S21 | 0 | 0 | 0 | 0 | 0 | 0 | 0 | 0 | 0 | 0 | 0 | 0 |
| mIGHV1-22 | 0.7 | 0.9 | 1.1 | 1.9 | 0.6 | 0.5 | 1.1 | 0.6 | 1.1 | 1.9 | 0.8 | 1.5 |
| mIGHV1S22 | 0 | 0 | 0 | 0 | 0 | 0 | 0 | 0 | 0 | 0 | 0 | 0 |
| mIGHV1-26 | 1.9 | 1.2 | 2.9 | 1.4 | 1.4 | 1 | 1.4 | 1.4 | 1.4 | 1.6 | 0.9 | 1.3 |
| mIGHV1S26 | 0.1 | 0.2 | 0 | 0 | 0 | 0 | 0 | 0 | 0 | 0 | 0 | 0.1 |
| mIGHV1S29 | 0 | 0 | 0 | 0 | 0 | 0 | 0 | 0 | 0 | 0 | 0 | 0 |
| mIGHV1-31 | 0 | 0 | 0 | 0 | 0 | 0 | 0 | 0 | 0 | 0 | 0 | 0 |
| mIGHV1S31 | 0 | 0 | 0 | 0 | 0 | 0 | 0 | 0 | 0 | 0 | 0 | 0 |
| mIGHV1S32 | 0 | 0 | 0 | 0 | 0 | 0 | 0 | 0 | 0 | 0 | 0 | 0 |
| mIGHV1S33 | 0 | 0 | 0 | 0 | 0 | 0 | 0 | 0 | 0 | 0 | 0 | 0 |
| mIGHV1-34 | 0.1 | 0.1 | 0 | 0.1 | 0.3 | 0.1 | 0.1 | 0.1 | 0.1 | 0.1 | 0.1 | 0.1 |
| mIGHV1S34 | 0 | 0 | 0 | 0 | 0 | 0 | 0 | 0 | 0 | 0 | 0 | 0 |
| mIGHV1S35 | 0 | 0 | 0 | 0 | 0 | 0 | 0 | 0 | 0 | 0 | 0 | 0 |
| mIGHV1-36 | 0.5 | 0.4 | 0.9 | 0.8 | 1.5 | 2.2 | 0.8 | 2.6 | 0.8 | 0.3 | 0.2 | 0.2 |
| mIGHV1S36 | 0 | 0 | 0 | 0 | 0 | 0 | 0 | 0 | 0 | 0 | 0 | 0 |
| mIGHV1-37 | 0 | 0 | 0.1 | 0 | 0 | 0 | 0 | 0 | 0 | 0.1 | 0.1 | 0 |
| mIGHV1S37 | 0 | 0 | 0 | 0 | 0 | 0 | 0 | 0 | 0 | 0 | 0 | 0 |
| mIGHV1-39 | 0.4 | 0.6 | 0.2 | 0.4 | 0.3 | 0.1 | 0.2 | 0.2 | 0.2 | 0.5 | 0.6 | 0.5 |
| mIGHV1S40 | 0 | 0 | 0 | 0 | 0 | 0 | 0.1 | 0 | 0.1 | 0 | 0 | 0 |
| mIGHV1S41 | 0 | 0 | 0 | 0 | 0 | 0 | 0 | 0 | 0 | 0 | 0 | 0 |
| mIGHV1-42 | 0.1 | 0 | 0.1 | 0.1 | 0.3 | 0 | 0.2 | 0.2 | 0.2 | 0.2 | 0.2 | 0.2 |
| mIGHV1-43 | 0 | 0 | 0 | 0 | 0 | 0 | 0 | 0 | 0 | 0 | 0 | 0 |
| mIGHV1S44 | 0 | 0 | 0 | 0 | 0 | 0 | 0 | 0 | 0 | 0 | 0 | 0 |
| mIGHV1S45 | 0 | 0 | 0 | 0 | 0 | 0 | 0 | 0 | 0 | 0 | 0 | 0 |
| mIGHV1S46 | 0 | 0 | 0 | 0 | 0 | 0 | 0 | 0 | 0 | 0 | 0 | 0 |
| mIGHV1-47 | 0 | 0 | 0 | 0 | 0 | 0 | 0 | 0 | 0 | 0 | 0 | 0 |
| mIGHV1-49 | 0 | 0 | 0 | 0 | 0 | 0 | 0 | 0 | 0 | 0 | 0 | 0 |
| mIGHV1S49 | 0 | 0 | 0 | 0 | 0 | 0 | 0 | 0 | 0 | 0 | 0 | 0 |
| mIGHV1-50 | 0.5 | 0.2 | 0.4 | 0.4 | 0.2 | 0.4 | 0.6 | 0.3 | 0.6 | 0.6 | 0.5 | 0.5 |
| mIGHV1S50 | 0 | 0 | 0 | 0 | 0 | 0 | 0 | 0 | 0 | 0 | 0 | 0 |
| mIGHV1-52 | 0 | 0 | 0.7 | 0 | 0 | 0 | 0 | 0 | 0 | 0.1 | 0 | 0 |
| mIGHV1S52 | 0 | 0 | 0 | 0 | 0 | 0 | 0 | 0 | 0 | 0 | 0 | 0 |
| mIGHV1-53 | 2.9 | 5.7 | 2.4 | 4.6 | 3.9 | 3.1 | 2.6 | 5 | 2.6 | 5.6 | 2.2 | 2.2 |
| mIGHV1S53 | 0 | 0 | 0 | 0 | 0 | 0 | 0 | 0 | 0 | 0 | 0 | 0 |
| mIGHV1-54 | 0.4 | 0.1 | 0.2 | 0.5 | 0.2 | 0.2 | 0.9 | 0 | 0.9 | 0.3 | 0.1 | 0.2 |
| mIGHV1-55 | 7.9 | 7 | 11.3 | 3.6 | 8.3 | 21.1 | 14.1 | 7 | 14.1 | 3.2 | 27.7 | 3.9 |
| mIGHV1S55 | 0 | 0 | 0 | 0 | 0 | 0 | 0 | 0 | 0 | 0 | 0 | 0 |
| mIGHV1-56 | 0 | 0 | 0 | 0 | 0 | 0 | 0 | 0 | 0 | 0 | 0 | 0 |
| mIGHV1S56 | 0 | 0 | 0 | 0 | 0 | 0 | 0 | 0 | 0 | 0 | 0 | 0 |
| mIGHV1-58 | 0 | 0.3 | 0.1 | 0.2 | 0.3 | 0.3 | 0.5 | 0.3 | 0.5 | 0.1 | 0 | 0.1 |
| mIGHV1-59 | 0.2 | 0.3 | 0.2 | 0.3 | 0.2 | 0.1 | 0.4 | 0 | 0.4 | 0.3 | 0.3 | 0.1 |
| mIGHV1-61 | 0.8 | 0.5 | 0.9 | 0.5 | 0.1 | 0.5 | 1 | 0.1 | 1 | 0.4 | 0.3 | 0.3 |
| mIGHV1S61 | 0 | 0 | 0 | 0 | 0 | 0 | 0 | 0 | 0 | 0 | 0 | 0 |
| mIGHV1-62-1 | 0 | 0 | 0 | 0 | 0 | 0 | 0 | 0 | 0 | 0 | 0 | 0 |
| mIGHV1-62-2 | 0 | 0 | 0 | 0 | 0 | 0 | 0 | 0 | 0 | 0 | 0 | 0 |
| mIGHV1-62-3 | 0 | 0 | 0 | 0 | 0 | 0 | 0 | 0 | 0 | 0 | 0 | 0 |
| mIGHV1-63 | 0.3 | 0.1 | 0.7 | 0.4 | 0.9 | 0.1 | 0.3 | 0.1 | 0.3 | 0 | 0.1 | 0.2 |
| mIGHV1-64 | 2.5 | 1.4 | 2 | 5.5 | 1.5 | 3.5 | 2.1 | 1.9 | 2.1 | 2 | 1.5 | 2.3 |
| mIGHV1S65 | 0 | 0 | 0 | 0 | 0 | 0 | 0 | 0 | 0 | 0 | 0 | 0 |
| mIGHV1-66 | 0.2 | 0.1 | 0.2 | 0.4 | 0.4 | 0.5 | 0.1 | 0.1 | 0.1 | 0.3 | 0.3 | 1.2 |
| mIGHV1-67 | 0 | 0 | 0 | 0 | 0 | 0 | 0 | 0 | 0 | 0 | 0 | 0 |
| mIGHV1S67 | 0 | 0 | 0 | 0 | 0 | 0 | 0 | 0 | 0 | 0 | 0 | 0 |
| mIGHV1S68 | 0 | 0 | 0 | 0 | 0 | 0 | 0 | 0 | 0 | 0 | 0 | 0 |
| mIGHV1-69 | 1.5 | 0.6 | 0.7 | 1 | 1.3 | 0.7 | 0.8 | 0.2 | 0.8 | 0.6 | 1.3 | 1.3 |
| mIGHV1S70 | 0 | 0 | 0 | 0 | 0 | 0 | 0 | 0 | 0 | 0 | 0 | 0 |
| mIGHV1-71 | 0.1 | 0.3 | 0.2 | 0.2 | 0.2 | 0.1 | 0.3 | 0.2 | 0.3 | 0.8 | 0.1 | 0.1 |
| mIGHV1-72 | 0.7 | 0.9 | 0.6 | 1.1 | 1.2 | 0.8 | 1.4 | 0.9 | 1.4 | 0.4 | 0.4 | 1 |
| mIGHV1S72 | 0 | 0 | 0 | 0 | 0 | 0 | 0 | 0 | 0 | 0 | 0 | 0 |
| mIGHV1S73 | 0 | 0 | 0 | 0 | 0 | 0 | 0 | 0 | 0 | 0 | 0 | 0 |
| mIGHV1-74 | 0.4 | 0.2 | 0.3 | 0.6 | 0.4 | 0.3 | 2 | 0.4 | 2 | 0.3 | 0.4 | 0 |
| mIGHV1-75 | 1.3 | 0.6 | 1.5 | 1.2 | 0.6 | 1.1 | 1.4 | 1 | 1.4 | 0.2 | 0.6 | 0.5 |
| mIGHV1S75 | 0 | 0 | 0 | 0 | 0 | 0 | 0 | 0 | 0 | 0 | 0 | 0 |
| mIGHV1-76 | 0.6 | 0.7 | 1.2 | 1.9 | 1.3 | 0.4 | 0.7 | 1.6 | 0.7 | 2.5 | 1.1 | 0.4 |
| mIGHV1-77 | 0.2 | 0 | 0.3 | 0.1 | 3.3 | 0 | 0.2 | 0.2 | 0.2 | 0 | 0.2 | 0.1 |
| mIGHV1-78 | 0.6 | 0.7 | 0.7 | 0.7 | 0.7 | 0.6 | 0.8 | 0.4 | 0.8 | 1.6 | 0.4 | 0.5 |
| mIGHV1S78 | 0 | 0 | 0 | 0 | 0 | 0 | 0 | 0 | 0 | 0 | 0 | 0 |
| mIGHV1-80 | 1 | 0.1 | 0.3 | 0.4 | 0.2 | 0.2 | 0.4 | 0.5 | 0.4 | 0.2 | 0.1 | 0.1 |
| mIGHV1-81 | 0 | 0 | 0 | 0 | 0 | 0 | 0 | 0 | 0 | 0 | 0 | 0 |
| mIGHV1S81 | 0 | 0 | 0 | 0 | 0 | 0 | 0 | 0 | 0 | 0 | 0 | 0 |
| mIGHV1-82 | 1 | 0.5 | 0.8 | 0.6 | 0.7 | 0.7 | 0.7 | 1.6 | 0.7 | 0.8 | 0.3 | 0.4 |
| mIGHV1S82 | 0 | 0 | 0 | 0 | 0 | 0 | 0 | 0 | 0 | 0 | 0 | 0 |
| mIGHV1S83 | 0 | 0 | 0 | 0 | 0 | 0 | 0 | 0 | 0 | 0 | 0 | 0 |
| mIGHV1-84 | 0 | 0 | 0.1 | 0.1 | 4.9 | 0.2 | 0.5 | 0.1 | 0.5 | 0 | 0 | 0.1 |
| mIGHV1-85 | 0.6 | 0.5 | 1.2 | 0.5 | 3.4 | 0.6 | 0.6 | 0.8 | 0.6 | 0.7 | 1.3 | 0.6 |
| mIGHV1S87 | 0 | 0 | 0 | 0 | 0 | 0 | 0 | 0 | 0 | 0 | 0 | 0 |
| mIGHV1S92 | 0 | 0 | 0 | 0 | 0 | 0 | 0 | 0 | 0 | 0 | 0 | 0 |
| mIGHV1S95 | 0 | 0 | 0 | 0 | 0 | 0 | 0 | 0 | 0 | 0 | 0 | 0 |
| mIGHV1S96 | 0 | 0 | 0 | 0 | 0 | 0 | 0 | 0 | 0 | 0 | 0 | 0 |
| mIGHV1S100 | 0 | 0 | 0 | 0 | 0 | 0 | 0 | 0 | 0 | 0 | 0 | 0 |
| mIGHV1S103 | 0 | 0 | 0 | 0 | 0 | 0 | 0 | 0 | 0 | 0 | 0 | 0 |
| mIGHV1S107 | 0 | 0 | 0 | 0 | 0 | 0 | 0 | 0 | 0 | 0 | 0 | 0 |
| mIGHV1S108 | 0 | 0 | 0 | 0 | 0 | 0 | 0 | 0 | 0 | 0 | 0 | 0 |
| mIGHV1S111 | 0 | 0 | 0 | 0 | 0 | 0 | 0 | 0 | 0 | 0 | 0 | 0 |
| mIGHV1S112 | 0 | 0 | 0 | 0 | 0 | 0 | 0 | 0 | 0 | 0 | 0 | 0 |
| mIGHV1S113 | 0 | 0 | 0 | 0 | 0 | 0 | 0 | 0 | 0 | 0 | 0 | 0 |
| mIGHV1S118 | 0 | 0 | 0 | 0 | 0 | 0 | 0 | 0 | 0 | 0 | 0 | 0 |
| mIGHV1S120 | 0 | 0 | 0 | 0 | 0 | 0 | 0 | 0 | 0 | 0 | 0 | 0 |
| mIGHV1S121 | 0 | 0 | 0 | 0 | 0 | 0 | 0 | 0 | 0 | 0 | 0 | 0 |
| mIGHV1S122 | 0 | 0 | 0 | 0 | 0 | 0 | 0 | 0 | 0 | 0 | 0 | 0 |
| mIGHV1S126 | 0 | 0 | 0 | 0 | 0 | 0 | 0 | 0 | 0 | 0 | 0 | 0 |

|  | **Peritoneal Cavity B-1a** | | | | | | | | | | | |
| --- | --- | --- | --- | --- | --- | --- | --- | --- | --- | --- | --- | --- |
| **VH Gene** | **WT 1** | **WT 2** | **WT 3** | **Unc93B1 1** | **Unc93B1 2** | **Unc93B1 3** | **TLR KO 1** | **TLR KO 2** | **TLR KO 3** | **2x4dKO 1** | **2x4dKO 2** | **2x4dKO 3** |
| mIGHV1S127 | 0 | 0 | 0 | 0 | 0 | 0 | 0 | 0 | 0 | 0 | 0 | 0 |
| mIGHV1S130 | 0 | 0 | 0 | 0 | 0 | 0 | 0 | 0 | 0 | 0 | 0 | 0 |
| mIGHV1S132 | 0 | 0 | 0 | 0 | 0 | 0 | 0 | 0 | 0 | 0 | 0 | 0 |
| mIGHV1S134 | 0 | 0 | 0 | 0 | 0 | 0 | 0 | 0 | 0 | 0 | 0 | 0 |
| mIGHV1S135 | 0 | 0 | 0 | 0 | 0 | 0 | 0 | 0 | 0 | 0 | 0 | 0 |
| mIGHV1S136 | 0 | 0 | 0 | 0 | 0 | 0 | 0 | 0 | 0 | 0 | 0 | 0 |
| mIGHV1S137 | 0 | 0 | 0 | 0 | 0 | 0 | 0 | 0 | 0 | 0 | 0 | 0 |
| mIGHV2-2-2 | 0 | 0 | 0 | 0 | 0 | 0 | 0 | 0 | 0 | 0 | 0 | 0 |
| mIGHV2-2 | 1.6 | 1 | 1.5 | 0.9 | 0.6 | 0.6 | 1 | 0.6 | 1 | 0.8 | 0.5 | 1 |
| mIGHV2-3-1 | 0 | 0 | 0 | 0 | 0 | 0 | 0 | 0 | 0 | 0 | 0 | 0 |
| mIGHV2-3 | 1.4 | 1.5 | 1.3 | 1.3 | 0.7 | 0.8 | 0.8 | 0.5 | 0.8 | 1.1 | 0.7 | 1.3 |
| mIGHV2S3 | 0 | 0 | 0 | 0 | 0 | 0 | 0 | 0 | 0 | 0 | 0 | 0 |
| mIGHV2-4-1 | 0 | 0 | 0 | 0 | 0 | 0 | 0 | 0 | 0 | 0 | 0 | 0 |
| mIGHV2-4 | 0.3 | 0 | 0 | 0.2 | 0.1 | 0 | 0.1 | 0.2 | 0.1 | 0.2 | 0.1 | 0 |
| mIGHV2-5-1 | 0.3 | 0.2 | 0.1 | 0.2 | 0.2 | 0.1 | 0.1 | 0.3 | 0.1 | 0.3 | 0.1 | 0.5 |
| mIGHV2-5 | 0 | 0 | 0 | 0 | 0 | 0 | 0 | 0 | 0 | 0 | 0 | 0 |
| mIGHV2-6-1 | 0 | 0 | 0 | 0 | 0 | 0 | 0 | 0 | 0 | 0 | 0 | 0 |
| mIGHV2-6-2 | 0.6 | 0.2 | 0.1 | 0.5 | 0.5 | 0.3 | 0.2 | 0.1 | 0.2 | 0.5 | 0.3 | 0.4 |
| mIGHV2-6-3 | 0 | 0 | 0 | 0 | 0 | 0 | 0 | 0 | 0 | 0 | 0 | 0 |
| mIGHV2-6-4 | 0 | 0 | 0 | 0 | 0 | 0 | 0 | 0 | 0 | 0 | 0 | 0 |
| mIGHV2-6-5 | 0 | 0 | 0 | 0 | 0 | 0 | 0 | 0 | 0 | 0 | 0 | 0 |
| mIGHV2-6-6 | 0 | 0 | 0 | 0 | 0 | 0 | 0 | 0 | 0 | 0 | 0 | 0 |
| mIGHV2-6-7 | 0 | 0 | 0 | 0 | 0 | 0 | 0 | 0 | 0 | 0 | 0 | 0 |
| mIGHV2-6-8 | 0.8 | 1.8 | 1 | 0.7 | 0.4 | 0.9 | 0.4 | 0.3 | 0.4 | 2 | 0.7 | 0.5 |
| mIGHV2-6 | 0 | 0 | 0 | 0 | 0 | 0 | 0 | 0 | 0 | 0 | 0 | 0 |
| mIGHV2-7 | 0 | 0 | 0 | 0 | 0 | 0 | 0 | 0 | 0 | 0 | 0 | 0 |
| mIGHV2-9-1 | 0 | 0 | 0 | 0 | 0 | 0 | 0 | 0 | 0 | 0 | 0 | 0 |
| mIGHV2-9 | 4.8 | 5.5 | 3.5 | 5.8 | 3.7 | 5.1 | 10.3 | 11.5 | 10.3 | 6.8 | 7.3 | 7.8 |
| mIGHV3-1 | 0 | 0 | 0 | 0 | 0 | 0 | 0 | 0 | 0 | 0 | 0 | 0 |
| mIGHV3S1 | 0 | 0 | 0 | 0 | 0 | 0 | 0 | 0 | 0 | 0 | 0 | 0 |
| mIGHV3-2 | 0 | 0 | 0 | 0 | 0.1 | 0 | 0 | 0 | 0 | 0 | 0 | 0 |
| mIGHV3-3 | 0.1 | 0 | 0.2 | 0 | 0 | 0 | 0 | 0.1 | 0 | 0 | 0 | 0 |
| mIGHV3-4 | 0 | 0 | 0 | 0 | 0 | 0 | 0 | 0 | 0 | 0 | 0 | 0 |
| mIGHV3-5 | 0 | 0 | 0 | 0 | 0 | 0 | 0 | 0.6 | 0 | 0 | 0 | 0 |
| mIGHV3-6 | 4.2 | 3.3 | 2.3 | 2.6 | 1.9 | 4 | 1.8 | 1.6 | 1.8 | 1.2 | 0.5 | 1.2 |
| mIGHV3S7 | 0 | 0 | 0 | 0 | 0 | 0 | 0 | 0 | 0 | 0 | 0 | 0 |
| mIGHV3-8 | 0.2 | 0.3 | 0.1 | 0.3 | 0.1 | 0.1 | 0.1 | 0.1 | 0.1 | 0.1 | 0.1 | 0.1 |
| mIGHV4-1 | 0 | 0 | 0 | 0 | 0 | 0 | 0 | 0 | 0 | 0 | 0 | 0 |
| mIGHV4-2 | 0 | 0 | 0 | 0 | 0 | 0 | 0 | 0 | 0 | 0 | 0 | 0 |
| mIGHV5-2 | 0 | 0.2 | 0 | 0.1 | 0 | 0 | 0 | 0 | 0 | 0.1 | 0 | 0 |
| mIGHV5-4 | 0.4 | 0.4 | 0.3 | 0.5 | 0.3 | 0.2 | 0.3 | 0.3 | 0.3 | 0.3 | 0.3 | 0.1 |
| mIGHV5S4 | 0 | 0 | 0 | 0 | 0 | 0 | 0 | 0 | 0 | 0 | 0 | 0 |
| mIGHV5-6-1 | 1.9 | 2.9 | 1.3 | 1.1 | 0.8 | 0.4 | 0.4 | 0.3 | 0.4 | 1 | 0.6 | 1 |
| mIGHV5-6-2 | 0 | 0 | 0 | 0 | 0 | 0 | 0 | 0 | 0 | 0 | 0 | 0 |
| mIGHV5-6-3 | 0 | 0 | 0 | 0 | 0 | 0 | 0 | 0 | 0 | 0 | 0 | 0 |
| mIGHV5-6-4 | 0 | 0 | 0 | 0 | 0 | 0 | 0 | 0 | 0 | 0 | 0 | 0 |
| mIGHV5-6-5 | 0 | 0 | 0 | 0 | 0 | 0 | 0 | 0 | 0 | 0 | 0 | 0 |
| mIGHV5-6-6 | 0 | 0 | 0 | 0 | 0 | 0 | 0 | 0 | 0 | 0 | 0 | 0 |
| mIGHV5-6 | 0 | 0 | 0.1 | 0 | 0 | 0 | 0 | 0 | 0 | 0 | 0 | 0 |
| mIGHV5-9-1 | 0.2 | 0.2 | 0.1 | 0.2 | 0.1 | 0.2 | 0.3 | 0.1 | 0.3 | 0.1 | 0.4 | 0.1 |
| mIGHV5-9-2 | 0 | 0 | 0 | 0 | 0 | 0 | 0 | 0 | 0 | 0 | 0 | 0 |
| mIGHV5-9-3 | 0 | 0 | 0 | 0 | 0 | 0 | 0 | 0 | 0 | 0 | 0 | 0 |
| mIGHV5-9-4 | 0 | 0 | 0 | 0 | 0 | 0 | 0 | 0 | 0 | 0 | 0 | 0 |
| mIGHV5-9-5 | 0 | 0 | 0 | 0 | 0 | 0 | 0 | 0 | 0 | 0 | 0 | 0 |
| mIGHV5-9 | 0.3 | 0.1 | 0.1 | 0.2 | 0.1 | 0 | 0.2 | 0 | 0.2 | 0.2 | 0 | 0.1 |
| mIGHV5S9 | 0 | 0 | 0 | 0 | 0 | 0 | 0 | 0 | 0 | 0 | 0 | 0 |
| mIGHV5-12-1 | 0 | 0 | 0 | 0 | 0 | 0 | 0 | 0 | 0 | 0 | 0 | 0 |
| mIGHV5-12-2 | 0 | 0 | 0 | 0 | 0 | 0 | 0 | 0 | 0 | 0 | 0 | 0 |
| mIGHV5-12-4 | 0 | 0 | 0 | 0 | 0 | 0 | 0 | 0 | 0 | 0 | 0 | 0 |
| mIGHV5-12 | 0 | 0.1 | 0 | 0.1 | 0.1 | 0.1 | 0.1 | 0 | 0.1 | 0.1 | 0 | 0 |
| mIGHV5S12 | 0 | 0 | 0 | 0 | 0 | 0 | 0 | 0 | 0 | 0 | 0 | 0 |
| mIGHV5-15 | 0.6 | 1.1 | 0.7 | 0.7 | 0.3 | 0.2 | 0.7 | 15.5 | 0.7 | 0.1 | 0 | 0 |
| mIGHV5-16 | 0.5 | 0.6 | 0.6 | 0.2 | 0.3 | 0.1 | 0.2 | 0.5 | 0.2 | 0.5 | 0.6 | 0.3 |
| mIGHV5-17 | 0.2 | 0.2 | 0.4 | 0.3 | 0.4 | 0.3 | 0.3 | 0.2 | 0.3 | 0.3 | 0.4 | 0.1 |
| mIGHV5S21 | 0 | 0 | 0 | 0 | 0 | 0 | 0 | 0 | 0 | 0 | 0 | 0 |
| mIGHV5S24 | 0 | 0 | 0 | 0 | 0 | 0 | 0 | 0 | 0 | 0 | 0 | 0 |
| mIGHV6S2 | 0 | 0 | 0 | 0 | 0 | 0 | 0 | 0 | 0 | 0 | 0 | 0 |
| mIGHV6-3 | 2 | 1.7 | 1.4 | 4 | 1.7 | 2.6 | 2.7 | 2.9 | 2.7 | 1.5 | 1.6 | 1.9 |
| mIGHV6S3 | 0 | 0 | 0 | 0 | 0 | 0 | 0 | 0 | 0 | 0 | 0 | 0 |
| mIGHV6-4 | 0 | 0 | 0 | 0 | 0 | 0 | 0 | 0 | 0 | 0 | 0 | 0 |
| mIGHV6S4 | 0 | 0 | 0 | 0 | 0 | 0 | 0 | 0 | 0 | 0 | 0 | 0 |
| mIGHV6-5 | 0 | 0 | 0 | 0 | 0 | 0 | 0 | 0 | 0 | 0 | 0 | 0 |
| mIGHV6-6 | 2.3 | 2.5 | 1.7 | 2.3 | 0.9 | 2.3 | 4.2 | 1.9 | 4.2 | 1.3 | 1.4 | 2.1 |
| mIGHV6-7 | 0 | 0 | 0 | 0 | 0 | 0 | 0 | 0 | 0 | 0 | 0 | 0 |
| mIGHV7-1 | 0.9 | 0.5 | 0.2 | 0.2 | 0.7 | 0.1 | 0.1 | 0.3 | 0.1 | 0.7 | 0.1 | 0.7 |
| mIGHV7-2 | 0 | 0 | 0 | 0 | 0 | 0 | 0 | 0 | 0 | 0 | 0 | 0 |
| mIGHV7-3 | 11.4 | 6.5 | 6.9 | 10.2 | 11.2 | 10 | 9.8 | 1.7 | 9.8 | 11.8 | 8.7 | 30.5 |
| mIGHV7-4 | 0.2 | 0.1 | 0.2 | 0.9 | 0.3 | 0.3 | 0.2 | 0.9 | 0.2 | 0.6 | 0.4 | 0.5 |
| mIGHV8-4 | 0 | 0 | 0 | 0 | 0 | 0 | 0 | 0 | 0 | 0 | 0 | 0 |
| mIGHV8-5 | 0.1 | 0 | 0 | 0.1 | 0.1 | 0 | 0 | 0 | 0 | 0 | 0 | 0.1 |
| mIGHV8-6 | 0 | 0 | 0 | 0 | 0 | 0 | 0 | 0 | 0 | 0 | 0 | 0 |
| mIGHV8-8 | 0.3 | 0.2 | 0.3 | 0.6 | 0.1 | 6 | 0.5 | 0.5 | 0.5 | 0.3 | 0.3 | 0.5 |
| mIGHV8S9 | 0 | 0 | 0 | 0 | 0 | 0 | 0 | 0 | 0 | 0 | 0 | 0 |
| mIGHV8-11 | 0 | 0 | 0 | 0 | 0 | 0 | 0 | 0 | 0 | 0 | 0 | 0 |
| mIGHV8-12 | 0.3 | 0.3 | 0.2 | 0.3 | 0.2 | 0.3 | 0.2 | 0.2 | 0.2 | 0.2 | 0.1 | 0.2 |
| mIGHV9-1 | 0.1 | 5 | 0.1 | 0.2 | 0.1 | 0.1 | 0.1 | 0 | 0.1 | 0 | 0 | 0 |
| mIGHV9-2-1 | 0 | 0 | 0 | 0 | 0 | 0 | 0 | 0 | 0 | 0 | 0 | 0 |
| mIGHV9-2 | 0.4 | 0.1 | 0.1 | 0.1 | 0 | 0.1 | 0.1 | 0 | 0.1 | 0 | 0 | 0 |
| mIGHV9-3-1 | 0 | 0 | 0 | 0 | 0 | 0 | 0 | 0 | 0 | 0 | 0 | 0 |
| mIGHV9-3 | 3.8 | 3.3 | 4 | 4.1 | 4.2 | 3.2 | 2.4 | 2.7 | 2.4 | 0.5 | 0.3 | 0.3 |
| mIGHV9-4 | 0.4 | 0.3 | 0.2 | 0.8 | 0.8 | 0.3 | 0.2 | 0.2 | 0.2 | 0 | 0 | 0 |
| mIGHV9S7 | 0 | 0 | 0 | 0 | 0 | 0 | 0 | 0 | 0 | 0 | 0 | 0 |
| mIGHV9S8 | 0 | 0 | 0 | 0 | 0 | 0 | 0 | 0 | 0 | 0 | 0 | 0 |
| mIGHV10-1 | 7.4 | 7.5 | 9.1 | 7.2 | 5.7 | 8.3 | 10.6 | 9.2 | 10.6 | 4.5 | 2.7 | 2.8 |
| mIGHV10-3 | 2 | 2.6 | 2.4 | 1.2 | 1.6 | 1.5 | 1.6 | 1.9 | 1.6 | 2.6 | 0.5 | 0.7 |
| mIGHV10S3 | 0 | 0 | 0 | 0 | 0 | 0 | 0 | 0 | 0 | 0 | 0 | 0 |
| mIGHV10S4 | 0 | 0 | 0 | 0 | 0 | 0 | 0 | 0 | 0 | 0 | 0 | 0 |
| mIGHV11-1 | 0 | 0 | 0 | 0 | 0 | 0 | 0 | 0 | 0 | 0 | 0.1 | 0.2 |
| mIGHV11-2 | 10.6 | 13 | 17.9 | 8.8 | 2.9 | 3.9 | 5.9 | 10.6 | 5.9 | 23.7 | 19.6 | 16.1 |
| mIGHV12-1-1 | 0 | 0 | 0 | 0 | 0 | 0 | 0 | 0 | 0 | 0 | 0 | 0 |
| mIGHV12-3 | 0.6 | 0.9 | 0.9 | 3.6 | 0 | 0.1 | 1.3 | 1.1 | 1.3 | 1.5 | 0 | 0.6 |
| mIGHV13-1 | 0 | 0 | 0 | 0 | 0 | 0 | 0 | 0 | 0 | 0 | 0 | 0 |
| mIGHV13-2 | 0 | 0 | 0 | 0.1 | 0 | 0 | 0.1 | 0 | 0.1 | 0 | 0 | 0.1 |
| mIGHV14-1 | 0.1 | 0 | 0.1 | 0.2 | 0.3 | 0 | 0.1 | 0 | 0.1 | 0 | 0.1 | 0.2 |
| mIGHV14-2 | 0.1 | 0 | 0 | 0 | 0.1 | 0 | 0.1 | 0 | 0.1 | 0 | 0 | 0 |
| mIGHV14-3 | 0.5 | 0.8 | 0.3 | 0.5 | 0.4 | 0.6 | 0.3 | 0.2 | 0.3 | 0.3 | 0.4 | 1.6 |
| mIGHV14-4 | 0.7 | 0.2 | 0.3 | 0.2 | 0.2 | 0.1 | 0.4 | 0.2 | 0.4 | 0.3 | 0.3 | 0.4 |
| mIGHV14S4 | 0 | 0 | 0 | 0 | 0 | 0 | 0 | 0 | 0 | 0 | 0 | 0 |
| mIGHV15-2 | 0 | 0 | 0 | 0 | 0 | 0 | 0 | 0 | 0 | 0 | 0 | 0 |
| mIGHV16-1 | 0 | 0 | 0 | 0 | 0 | 0 | 0 | 0 | 0 | 0 | 0 | 0 |

|  | **Spleen B-1a** | | | | | | | | | | | |
| --- | --- | --- | --- | --- | --- | --- | --- | --- | --- | --- | --- | --- |
| **VH Gene** | **WT 1** | **WT 2** | **WT 3** | **Unc93B1 1** | **Unc93B1 2** | **Unc93B1 3** | **TLR KO 1** | **TLR KO 2** | **TLR KO 3** | **2x4dKO 1** | **2x4dKO 2** | **2x4dKO 3** |
| mIGHV1-4 | 0.6 | 0.7 | 0.3 | 0.8 | 7.6 | 1.2 | 0.2 | 1.9 | 0.8 | 1.3 | 0.7 | 1.1 |
| mIGHV1-5 | 0.1 | 0.4 | 0.2 | 0.4 | 0.7 | 0.2 | 0.1 | 3.2 | 0.2 | 0.4 | 0 | 0 |
| mIGHV1S5 | 0 | 0 | 0 | 0 | 0 | 0 | 0 | 0 | 0 | 0 | 0 | 0 |
| mIGHV1-7 | 1.7 | 1 | 1.1 | 1.4 | 1.4 | 1.4 | 0.9 | 1.2 | 0.7 | 1.3 | 0.9 | 3.5 |
| mIGHV1-9 | 1 | 2.7 | 0.7 | 2 | 1.6 | 1.4 | 1.1 | 1.7 | 1 | 1.4 | 1 | 0 |
| mIGHV1-11 | 0.1 | 0 | 0 | 0 | 0 | 0 | 0 | 0 | 0.1 | 0 | 0 | 0.2 |
| mIGHV1-12 | 0.1 | 0.1 | 0.1 | 0.1 | 0.1 | 0.2 | 0 | 0.2 | 0.1 | 0 | 0.1 | 0 |
| mIGHV1S12 | 0 | 0 | 0 | 0 | 0 | 0 | 0 | 0 | 0 | 0 | 0 | 0 |
| mIGHV1-14 | 0 | 0 | 0 | 0 | 0 | 0 | 0 | 0 | 0 | 0 | 0 | 0 |
| mIGHV1S14 | 0 | 0 | 0 | 0 | 0 | 0 | 0 | 0 | 0 | 0 | 0 | 0 |
| mIGHV1-15 | 0 | 0 | 0 | 0 | 0 | 0 | 0 | 0 | 0 | 0 | 0 | 0 |
| mIGHV1-17-1 | 0 | 0 | 0 | 0 | 0 | 0 | 0 | 0 | 0 | 0 | 0 | 0 |
| mIGHV1-18 | 0.9 | 1 | 0.6 | 1.4 | 0.8 | 0.8 | 0.4 | 0.6 | 0.6 | 1.5 | 0.7 | 2.2 |
| mIGHV1-19 | 3.1 | 2.8 | 2 | 1.7 | 1.5 | 1.7 | 1.2 | 1.2 | 1.6 | 2.1 | 1.8 | 2.2 |
| mIGHV1-20 | 0.2 | 0.2 | 0 | 0.1 | 0.1 | 0.1 | 0.2 | 0.2 | 0.2 | 0.6 | 0.1 | 0 |
| mIGHV1S20 | 0 | 0 | 0 | 0 | 0 | 0 | 0 | 0 | 0 | 0 | 0 | 0 |
| mIGHV1S21 | 0 | 0 | 0 | 0 | 0 | 0 | 0 | 0 | 0 | 0 | 0 | 0 |
| mIGHV1-22 | 0.6 | 0.6 | 0.6 | 1.7 | 0.6 | 0.5 | 0.4 | 0.8 | 2.1 | 1.6 | 1.1 | 1.3 |
| mIGHV1S22 | 0 | 0 | 0 | 0 | 0 | 0 | 0 | 0 | 0 | 0 | 0 | 0 |
| mIGHV1-26 | 2.9 | 3.1 | 1.7 | 3.1 | 1.9 | 2.9 | 0.8 | 2.6 | 1.5 | 2.8 | 2.4 | 3.3 |
| mIGHV1S26 | 0 | 0 | 0 | 0 | 0 | 0 | 0 | 0 | 0 | 0 | 0 | 0 |
| mIGHV1S29 | 0 | 0 | 0 | 0 | 0 | 0 | 0 | 0 | 0 | 0 | 0 | 0 |
| mIGHV1-31 | 0 | 0 | 0 | 0 | 0 | 0 | 0 | 0 | 0 | 0 | 0 | 0 |
| mIGHV1S31 | 0 | 0 | 0 | 0 | 0 | 0 | 0 | 0 | 0 | 0 | 0 | 0 |
| mIGHV1S32 | 0 | 0 | 0 | 0 | 0 | 0 | 0 | 0 | 0 | 0 | 0 | 0 |
| mIGHV1S33 | 0 | 0 | 0 | 0 | 0 | 0 | 0 | 0 | 0 | 0 | 0 | 0 |
| mIGHV1-34 | 0.2 | 0.1 | 0.1 | 0.4 | 0.2 | 0.1 | 0.2 | 0.1 | 0 | 0.3 | 0.1 | 0.8 |
| mIGHV1S34 | 0 | 0 | 0 | 0 | 0 | 0 | 0 | 0 | 0 | 0 | 0 | 0 |
| mIGHV1S35 | 0 | 0 | 0 | 0 | 0 | 0 | 0 | 0 | 0 | 0 | 0 | 0 |
| mIGHV1-36 | 0.4 | 0.4 | 0.6 | 0.5 | 0.8 | 2.5 | 0.2 | 3.6 | 0.7 | 0.6 | 0.1 | 0.4 |
| mIGHV1S36 | 0 | 0 | 0 | 0 | 0 | 0 | 0 | 0 | 0 | 0 | 0 | 0 |
| mIGHV1-37 | 0.1 | 0 | 0 | 0.1 | 0 | 0 | 0 | 0 | 0.1 | 0 | 0 | 0 |
| mIGHV1S37 | 0 | 0 | 0 | 0 | 0 | 0 | 0 | 0 | 0 | 0 | 0 | 0 |
| mIGHV1-39 | 0.2 | 0.5 | 0.1 | 0.3 | 0.3 | 0.2 | 0.3 | 0.3 | 0.1 | 0.3 | 0.2 | 0.8 |
| mIGHV1S40 | 0 | 0 | 0 | 0 | 0 | 0 | 0 | 0 | 0 | 0 | 0 | 0 |
| mIGHV1S41 | 0 | 0 | 0 | 0 | 0 | 0 | 0 | 0 | 0 | 0 | 0 | 0 |
| mIGHV1-42 | 0.5 | 0.5 | 0.3 | 1.2 | 0.2 | 0.2 | 0.2 | 0.2 | 0.3 | 0.3 | 0.2 | 0.2 |
| mIGHV1-43 | 0 | 0 | 0 | 0 | 0 | 0 | 0 | 0 | 0 | 0 | 0 | 0 |
| mIGHV1S44 | 0 | 0 | 0 | 0 | 0 | 0 | 0 | 0 | 0 | 0 | 0 | 0 |
| mIGHV1S45 | 0 | 0 | 0 | 0 | 0 | 0 | 0 | 0 | 0 | 0 | 0 | 0 |
| mIGHV1S46 | 0 | 0 | 0 | 0 | 0 | 0 | 0 | 0 | 0 | 0 | 0 | 0 |
| mIGHV1-47 | 0 | 0 | 0 | 0 | 0 | 0 | 0 | 0 | 0 | 0 | 0 | 0 |
| mIGHV1-49 | 0 | 0 | 0 | 0 | 0 | 0 | 0 | 0 | 0 | 0 | 0 | 0 |
| mIGHV1S49 | 0 | 0 | 0 | 0 | 0 | 0 | 0 | 0 | 0 | 0 | 0 | 0 |
| mIGHV1-50 | 0.4 | 0.8 | 0.4 | 0.8 | 0.5 | 0.6 | 0.3 | 1 | 0.5 | 1.2 | 0.3 | 1.3 |
| mIGHV1S50 | 0 | 0 | 0 | 0 | 0 | 0 | 0 | 0 | 0 | 0 | 0 | 0 |
| mIGHV1-52 | 0 | 0 | 0.2 | 0 | 0 | 0.1 | 0 | 0 | 0 | 0 | 0 | 0 |
| mIGHV1S52 | 0 | 0 | 0 | 0 | 0 | 0 | 0 | 0 | 0 | 0 | 0 | 0 |
| mIGHV1-53 | 3.9 | 3.3 | 1.8 | 4.4 | 7.4 | 4.5 | 2 | 6.2 | 2.4 | 6.9 | 3.5 | 1.6 |
| mIGHV1S53 | 0 | 0 | 0 | 0 | 0 | 0 | 0 | 0 | 0 | 0 | 0 | 0 |
| mIGHV1-54 | 1.1 | 0.3 | 0.2 | 0.5 | 0.2 | 0.2 | 0.1 | 0 | 0.4 | 0 | 0.4 | 0.4 |
| mIGHV1-55 | 9.1 | 5.8 | 34.2 | 6.5 | 18.2 | 8.1 | 7 | 11.5 | 14.5 | 4.7 | 7.6 | 14.9 |
| mIGHV1S55 | 0 | 0 | 0 | 0 | 0 | 0 | 0 | 0 | 0 | 0 | 0 | 0 |
| mIGHV1-56 | 0 | 0 | 0 | 0 | 0 | 0 | 0 | 0 | 0 | 0 | 0 | 0 |
| mIGHV1S56 | 0 | 0 | 0 | 0 | 0 | 0 | 0 | 0 | 0 | 0 | 0 | 0 |
| mIGHV1-58 | 0.5 | 0.5 | 0.2 | 0.7 | 0.6 | 0.4 | 1 | 0.9 | 0.3 | 0.6 | 0.3 | 0.3 |
| mIGHV1-59 | 0.4 | 0.2 | 0.3 | 0.5 | 0.4 | 0.3 | 0.2 | 0.3 | 0.3 | 0.3 | 0.1 | 0 |
| mIGHV1-61 | 0.4 | 0.5 | 0.6 | 1 | 1.6 | 1.1 | 0.2 | 0.3 | 1.1 | 0.8 | 1 | 0 |
| mIGHV1S61 | 0 | 0 | 0 | 0 | 0 | 0 | 0 | 0 | 0 | 0 | 0 | 0 |
| mIGHV1-62-1 | 0 | 0 | 0 | 0 | 0 | 0 | 0 | 0 | 0 | 0 | 0 | 0 |
| mIGHV1-62-2 | 0 | 0 | 0 | 0 | 0 | 0 | 0 | 0 | 0 | 0 | 0 | 0 |
| mIGHV1-62-3 | 0 | 0 | 0 | 0 | 0 | 0 | 0 | 0 | 0 | 0 | 0 | 0 |
| mIGHV1-63 | 0.2 | 0.1 | 0 | 0.3 | 0.2 | 0 | 1.1 | 0.2 | 0.3 | 0 | 0.1 | 0 |
| mIGHV1-64 | 4.4 | 4.4 | 2.9 | 4.6 | 3 | 4.1 | 2.7 | 6.3 | 3.2 | 4.1 | 4.4 | 0.9 |
| mIGHV1S65 | 0 | 0 | 0 | 0 | 0 | 0 | 0 | 0 | 0 | 0 | 0 | 0 |
| mIGHV1-66 | 0.9 | 0.6 | 0.3 | 0.8 | 0.7 | 0.7 | 0.2 | 0.2 | 0.3 | 1.1 | 0.4 | 1.9 |
| mIGHV1-67 | 0 | 0 | 0 | 0 | 0 | 0 | 0 | 0 | 0 | 0 | 0 | 0 |
| mIGHV1S67 | 0 | 0 | 0 | 0 | 0 | 0 | 0 | 0 | 0 | 0 | 0 | 0 |
| mIGHV1S68 | 0 | 0 | 0 | 0 | 0 | 0 | 0 | 0 | 0 | 0 | 0 | 0 |
| mIGHV1-69 | 1.7 | 1.6 | 0.8 | 2.2 | 1.3 | 2 | 0.7 | 1.4 | 0.8 | 3.3 | 0.9 | 1.5 |
| mIGHV1S70 | 0 | 0 | 0 | 0 | 0 | 0 | 0 | 0 | 0 | 0 | 0 | 0 |
| mIGHV1-71 | 0.4 | 0.5 | 0.1 | 0.3 | 0.3 | 0.2 | 0.1 | 0.1 | 0.7 | 0.4 | 0.1 | 0 |
| mIGHV1-72 | 1.7 | 1 | 0.8 | 1.3 | 0.9 | 1.9 | 0.3 | 1.2 | 1.2 | 0.1 | 1.2 | 1.6 |
| mIGHV1S72 | 0 | 0 | 0 | 0 | 0 | 0 | 0 | 0 | 0 | 0 | 0 | 0 |
| mIGHV1S73 | 0 | 0 | 0 | 0 | 0 | 0 | 0 | 0 | 0 | 0 | 0 | 0 |
| mIGHV1-74 | 0.8 | 0.3 | 0.6 | 0.6 | 0.8 | 0.9 | 0.2 | 1.5 | 5.8 | 0.5 | 0.4 | 0.9 |
| mIGHV1-75 | 1.7 | 3.3 | 2.4 | 1.8 | 1.3 | 1.3 | 0.5 | 2.8 | 2.6 | 2.2 | 1.6 | 1.6 |
| mIGHV1S75 | 0 | 0 | 0 | 0 | 0 | 0 | 0 | 0 | 0 | 0 | 0 | 0 |
| mIGHV1-76 | 1.1 | 0.9 | 0.4 | 1.7 | 0.5 | 1 | 0.8 | 0.6 | 0.5 | 0.7 | 0.2 | 0.1 |
| mIGHV1-77 | 0.2 | 0.1 | 0.1 | 0.2 | 0.6 | 0.1 | 0.1 | 0.3 | 0.2 | 0.4 | 0 | 0 |
| mIGHV1-78 | 0.8 | 1 | 0.7 | 1.3 | 0.6 | 0.7 | 0.3 | 0.5 | 0.3 | 1.3 | 0.7 | 0.9 |
| mIGHV1S78 | 0 | 0 | 0 | 0 | 0 | 0 | 0 | 0 | 0 | 0 | 0 | 0 |
| mIGHV1-80 | 0.6 | 0.5 | 0.3 | 0.7 | 0.4 | 8.7 | 0.1 | 0.4 | 0.1 | 0.5 | 0.3 | 0.2 |
| mIGHV1-81 | 0 | 0 | 0 | 0 | 0 | 0 | 0 | 0 | 0 | 0 | 0 | 0 |
| mIGHV1S81 | 0 | 0 | 0 | 0 | 0 | 0 | 0 | 0 | 0 | 0 | 0 | 0 |
| mIGHV1-82 | 1 | 0.7 | 0.3 | 1.1 | 1.4 | 1 | 0.2 | 0.9 | 0.4 | 0.5 | 0.5 | 0.4 |
| mIGHV1S82 | 0 | 0 | 0 | 0 | 0 | 0 | 0 | 0 | 0 | 0 | 0 | 0 |
| mIGHV1S83 | 0 | 0 | 0 | 0 | 0 | 0 | 0 | 0 | 0 | 0 | 0 | 0 |
| mIGHV1-84 | 0.1 | 0.3 | 0.2 | 0.2 | 1.5 | 0.3 | 0.1 | 0.2 | 13.2 | 0.7 | 0 | 0 |
| mIGHV1-85 | 1.5 | 1.1 | 0.9 | 1.1 | 1.9 | 1.3 | 0.4 | 0.5 | 2.4 | 0.9 | 0.8 | 1.3 |
| mIGHV1S87 | 0 | 0 | 0 | 0 | 0 | 0 | 0 | 0 | 0 | 0 | 0 | 0 |
| mIGHV1S92 | 0 | 0 | 0 | 0 | 0 | 0 | 0 | 0 | 0 | 0 | 0 | 0 |
| mIGHV1S95 | 0 | 0 | 0 | 0 | 0 | 0 | 0 | 0 | 0 | 0 | 0 | 0 |
| mIGHV1S96 | 0 | 0 | 0 | 0 | 0 | 0 | 0 | 0 | 0 | 0 | 0 | 0 |
| mIGHV1S100 | 0 | 0 | 0 | 0 | 0 | 0 | 0 | 0 | 0 | 0 | 0 | 0 |
| mIGHV1S103 | 0 | 0 | 0 | 0 | 0 | 0 | 0 | 0 | 0 | 0 | 0 | 0 |
| mIGHV1S107 | 0 | 0 | 0 | 0 | 0 | 0 | 0 | 0 | 0 | 0 | 0 | 0 |
| mIGHV1S108 | 0 | 0 | 0 | 0 | 0 | 0 | 0 | 0 | 0 | 0 | 0 | 0 |
| mIGHV1S111 | 0 | 0 | 0 | 0 | 0 | 0 | 0 | 0 | 0 | 0 | 0 | 0 |
| mIGHV1S112 | 0 | 0 | 0 | 0 | 0 | 0 | 0 | 0 | 0 | 0 | 0 | 0 |
| mIGHV1S113 | 0 | 0 | 0 | 0 | 0 | 0 | 0 | 0 | 0 | 0 | 0 | 0 |
| mIGHV1S118 | 0 | 0 | 0 | 0 | 0 | 0 | 0 | 0 | 0 | 0 | 0 | 0 |
| mIGHV1S120 | 0 | 0 | 0 | 0 | 0 | 0 | 0 | 0 | 0 | 0 | 0 | 0 |
| mIGHV1S121 | 0 | 0 | 0 | 0 | 0 | 0 | 0 | 0 | 0 | 0 | 0 | 0 |
| mIGHV1S122 | 0 | 0 | 0 | 0 | 0 | 0 | 0 | 0 | 0 | 0 | 0 | 0 |
| mIGHV1S126 | 0 | 0 | 0 | 0 | 0 | 0 | 0 | 0 | 0 | 0 | 0 | 0 |

|  | **Spleen B-1a** | | | | | | | | | | | |
| --- | --- | --- | --- | --- | --- | --- | --- | --- | --- | --- | --- | --- |
| **VH Gene** | **WT 1** | **WT 2** | **WT 3** | **Unc93B1 1** | **Unc93B1 2** | **Unc93B1 3** | **TLR KO 1** | **TLR KO 2** | **TLR KO 3** | **2x4dKO 1** | **2x4dKO 2** | **2x4dKO 3** |
| mIGHV1S127 | 0 | 0 | 0 | 0 | 0 | 0 | 0 | 0 | 0 | 0 | 0 | 0 |
| mIGHV1S130 | 0 | 0 | 0 | 0 | 0 | 0 | 0 | 0 | 0 | 0 | 0 | 0 |
| mIGHV1S132 | 0 | 0 | 0 | 0 | 0 | 0 | 0 | 0 | 0 | 0 | 0 | 0 |
| mIGHV1S134 | 0 | 0 | 0 | 0 | 0 | 0 | 0 | 0 | 0 | 0 | 0 | 0 |
| mIGHV1S135 | 0 | 0 | 0 | 0 | 0 | 0 | 0 | 0 | 0 | 0 | 0 | 0 |
| mIGHV1S136 | 0 | 0 | 0 | 0 | 0 | 0 | 0 | 0 | 0 | 0 | 0 | 0 |
| mIGHV1S137 | 0 | 0 | 0 | 0 | 0 | 0 | 0 | 0 | 0 | 0 | 0 | 0 |
| mIGHV2-2-2 | 0 | 0 | 0 | 0 | 0 | 0 | 0 | 0 | 0 | 0 | 0 | 0 |
| mIGHV2-2 | 1.6 | 0.7 | 1 | 0.6 | 0.6 | 1.1 | 0.2 | 0.7 | 0.6 | 0.5 | 0.4 | 0 |
| mIGHV2-3-1 | 0 | 0 | 0 | 0 | 0 | 0 | 0 | 0 | 0 | 0 | 0 | 0 |
| mIGHV2-3 | 1.2 | 1 | 0.8 | 1.1 | 1.1 | 1.1 | 0.6 | 1.2 | 0.7 | 1.6 | 1.1 | 0.7 |
| mIGHV2S3 | 0 | 0 | 0 | 0 | 0 | 0 | 0 | 0 | 0 | 0 | 0 | 0 |
| mIGHV2-4-1 | 0 | 0 | 0 | 0 | 0 | 0 | 0 | 0 | 0 | 0 | 0 | 0 |
| mIGHV2-4 | 0.1 | 0.1 | 0 | 0.1 | 0.1 | 0.1 | 0 | 0 | 0 | 0 | 0 | 0 |
| mIGHV2-5-1 | 0.2 | 0.2 | 0.1 | 0.2 | 0.2 | 0.1 | 0 | 0.2 | 0 | 0.1 | 0.3 | 0 |
| mIGHV2-5 | 0 | 0 | 0 | 0 | 0 | 0 | 0 | 0 | 0 | 0 | 0 | 0 |
| mIGHV2-6-1 | 0 | 0 | 0 | 0 | 0 | 0 | 0 | 0 | 0 | 0 | 0 | 0 |
| mIGHV2-6-2 | 0.3 | 0.5 | 0.3 | 0.9 | 1.4 | 0.5 | 0.2 | 0.3 | 0.1 | 0 | 0.7 | 0.8 |
| mIGHV2-6-3 | 0 | 0 | 0 | 0 | 0 | 0 | 0 | 0 | 0 | 0 | 0 | 0 |
| mIGHV2-6-4 | 0 | 0 | 0 | 0 | 0 | 0 | 0 | 0 | 0 | 0 | 0 | 0 |
| mIGHV2-6-5 | 0 | 0 | 0 | 0 | 0 | 0 | 0 | 0 | 0 | 0 | 0 | 0 |
| mIGHV2-6-6 | 0 | 0 | 0 | 0 | 0 | 0 | 0 | 0 | 0 | 0 | 0 | 0 |
| mIGHV2-6-7 | 0 | 0 | 0 | 0 | 0 | 0 | 0 | 0 | 0 | 0 | 0 | 0 |
| mIGHV2-6-8 | 0.5 | 0.5 | 0.1 | 0.3 | 0.4 | 0.5 | 0.1 | 0 | 0.1 | 0.6 | 0.4 | 0.2 |
| mIGHV2-6 | 0 | 0 | 0 | 0 | 0 | 0 | 0 | 0 | 0 | 0 | 0 | 0 |
| mIGHV2-7 | 0 | 0 | 0 | 0 | 0 | 0 | 0 | 0 | 0 | 0 | 0 | 0 |
| mIGHV2-9-1 | 0 | 0 | 0 | 0 | 0 | 0 | 0 | 0 | 0 | 0 | 0 | 0 |
| mIGHV2-9 | 3.1 | 3.5 | 1.6 | 4.2 | 3.2 | 7.5 | 2.5 | 7.4 | 3.3 | 4.8 | 2.1 | 6.3 |
| mIGHV3-1 | 0 | 0 | 0 | 0 | 0 | 0 | 0 | 0 | 0 | 0 | 0 | 0 |
| mIGHV3S1 | 0 | 0 | 0 | 0 | 0 | 0 | 0 | 0 | 0 | 0 | 0 | 0 |
| mIGHV3-2 | 0 | 0 | 0 | 0 | 0 | 0 | 0 | 0 | 0 | 0 | 0 | 0 |
| mIGHV3-3 | 0.1 | 0 | 0 | 0 | 0.1 | 0 | 0.1 | 0 | 0 | 0 | 0 | 0 |
| mIGHV3-4 | 0 | 0 | 0 | 0 | 0 | 0 | 0 | 0 | 0 | 0 | 0 | 0 |
| mIGHV3-5 | 0.1 | 0 | 0.1 | 0 | 0 | 0 | 0 | 0.1 | 0 | 0 | 0 | 0 |
| mIGHV3-6 | 3.1 | 2.8 | 2.2 | 3.1 | 2.5 | 3.4 | 1.2 | 2.3 | 3.3 | 2 | 1.1 | 1.2 |
| mIGHV3S7 | 0 | 0 | 0 | 0 | 0 | 0 | 0 | 0 | 0 | 0 | 0 | 0 |
| mIGHV3-8 | 0.2 | 0.2 | 0.1 | 0.1 | 0.2 | 0.2 | 0 | 0.3 | 0.1 | 0.1 | 0.1 | 0.1 |
| mIGHV4-1 | 0 | 0 | 0 | 0 | 0 | 0 | 0 | 0 | 0 | 0 | 0 | 0 |
| mIGHV4-2 | 0 | 0 | 0 | 0 | 0 | 0 | 0 | 0 | 0 | 0 | 0 | 0 |
| mIGHV5-2 | 0 | 0.2 | 0 | 0 | 0 | 0.1 | 0 | 0.1 | 0 | 0 | 0.1 | 0 |
| mIGHV5-4 | 0.4 | 0.4 | 0.1 | 0.4 | 0.2 | 0.2 | 0.1 | 0 | 0.2 | 0.2 | 0.3 | 0.2 |
| mIGHV5S4 | 0 | 0 | 0 | 0 | 0 | 0 | 0 | 0 | 0 | 0 | 0 | 0 |
| mIGHV5-6-1 | 1.1 | 1.1 | 0.6 | 0.9 | 0.4 | 0.6 | 0.1 | 0.6 | 0.6 | 0.9 | 0.6 | 0.9 |
| mIGHV5-6-2 | 0 | 0 | 0 | 0 | 0 | 0 | 0 | 0 | 0 | 0 | 0 | 0 |
| mIGHV5-6-3 | 0 | 0 | 0 | 0 | 0 | 0 | 0 | 0 | 0 | 0 | 0 | 0 |
| mIGHV5-6-4 | 0 | 0 | 0 | 0 | 0 | 0 | 0 | 0 | 0 | 0 | 0 | 0 |
| mIGHV5-6-5 | 0 | 0 | 0 | 0 | 0 | 0 | 0 | 0 | 0 | 0 | 0 | 0 |
| mIGHV5-6-6 | 0 | 0 | 0 | 0 | 0 | 0 | 0 | 0 | 0 | 0 | 0 | 0 |
| mIGHV5-6 | 0.1 | 0.1 | 0 | 0 | 0.3 | 0.1 | 0 | 0.1 | 0.1 | 0 | 0 | 0.2 |
| mIGHV5-9-1 | 0.8 | 0.8 | 0.1 | 0.2 | 0.1 | 0.2 | 0 | 0 | 0.2 | 0.1 | 0 | 0.1 |
| mIGHV5-9-2 | 0 | 0 | 0 | 0 | 0 | 0 | 0 | 0 | 0 | 0 | 0 | 0 |
| mIGHV5-9-3 | 0 | 0 | 0 | 0 | 0 | 0 | 0 | 0 | 0 | 0 | 0 | 0 |
| mIGHV5-9-4 | 0 | 0 | 0 | 0 | 0 | 0 | 0 | 0 | 0 | 0 | 0 | 0 |
| mIGHV5-9-5 | 0 | 0 | 0 | 0 | 0 | 0 | 0 | 0 | 0 | 0 | 0 | 0 |
| mIGHV5-9 | 0.1 | 0.1 | 0.1 | 0.1 | 0.4 | 0.1 | 0.1 | 0 | 0.1 | 0.2 | 0.1 | 0 |
| mIGHV5S9 | 0 | 0 | 0 | 0 | 0 | 0 | 0 | 0 | 0 | 0 | 0 | 0 |
| mIGHV5-12-1 | 0 | 0 | 0 | 0 | 0 | 0 | 0 | 0 | 0 | 0 | 0 | 0 |
| mIGHV5-12-2 | 0 | 0 | 0 | 0 | 0 | 0 | 0 | 0 | 0 | 0 | 0 | 0 |
| mIGHV5-12-4 | 0 | 0 | 0 | 0 | 0 | 0 | 0 | 0 | 0 | 0 | 0 | 0 |
| mIGHV5-12 | 0 | 0.1 | 0 | 0.1 | 0 | 0.1 | 0.1 | 0.2 | 0 | 0.1 | 0 | 0 |
| mIGHV5S12 | 0 | 0 | 0 | 0 | 0 | 0 | 0 | 0 | 0 | 0 | 0 | 0 |
| mIGHV5-15 | 0.1 | 0.2 | 0.2 | 0.4 | 0.3 | 0.3 | 0.2 | 3.2 | 1.2 | 0.1 | 0.1 | 0 |
| mIGHV5-16 | 0.5 | 0.8 | 0.2 | 0.3 | 0.4 | 0.3 | 3.5 | 0.7 | 0.1 | 0.6 | 0.1 | 0.1 |
| mIGHV5-17 | 0.3 | 0.6 | 0.5 | 0.5 | 0.3 | 0.4 | 0.3 | 0.4 | 0.2 | 0.6 | 0.2 | 0 |
| mIGHV5S21 | 0 | 0 | 0 | 0 | 0 | 0 | 0 | 0 | 0 | 0 | 0 | 0 |
| mIGHV5S24 | 0 | 0 | 0 | 0 | 0 | 0 | 0 | 0 | 0 | 0 | 0 | 0 |
| mIGHV6S2 | 0 | 0 | 0 | 0 | 0 | 0 | 0 | 0 | 0 | 0 | 0 | 0 |
| mIGHV6-3 | 4.2 | 2.8 | 2.5 | 3.1 | 2.1 | 1.9 | 1.8 | 2.5 | 7.1 | 3.1 | 4 | 4.3 |
| mIGHV6S3 | 0 | 0 | 0 | 0 | 0 | 0 | 0 | 0 | 0 | 0 | 0 | 0 |
| mIGHV6-4 | 0.1 | 0 | 0 | 0 | 0 | 0 | 0 | 0 | 0 | 0 | 0 | 0 |
| mIGHV6S4 | 0 | 0 | 0 | 0 | 0 | 0 | 0 | 0 | 0 | 0 | 0 | 0 |
| mIGHV6-5 | 0 | 0 | 0 | 0 | 0 | 0 | 0 | 0 | 0 | 0 | 0 | 0 |
| mIGHV6-6 | 2.8 | 1.6 | 1.5 | 2.6 | 0.9 | 1.6 | 1.1 | 2.6 | 1.9 | 1.4 | 2.3 | 1.9 |
| mIGHV6-7 | 0 | 0 | 0 | 0 | 0 | 0 | 0 | 0 | 0 | 0 | 0 | 0 |
| mIGHV7-1 | 0.7 | 0.1 | 0.4 | 0.2 | 0.4 | 0.2 | 0.2 | 0 | 0.1 | 0.2 | 0.3 | 0.2 |
| mIGHV7-2 | 0 | 0 | 0 | 0 | 0 | 0 | 0 | 0 | 0 | 0 | 0 | 0 |
| mIGHV7-3 | 6.1 | 4.7 | 2.5 | 9.9 | 4.3 | 5.6 | 50.3 | 2.4 | 4.2 | 11.4 | 33.1 | 11.1 |
| mIGHV7-4 | 0.1 | 0.2 | 0.1 | 0.3 | 0.4 | 0.7 | 0.2 | 0.4 | 0.2 | 0.5 | 0.1 | 0 |
| mIGHV8-4 | 0 | 0 | 0 | 0 | 0 | 0 | 0 | 0 | 0 | 0 | 0 | 0 |
| mIGHV8-5 | 0.2 | 0.1 | 0 | 0.2 | 0.1 | 0.1 | 0 | 0.1 | 0 | 0.1 | 0.1 | 0.4 |
| mIGHV8-6 | 0 | 0 | 0 | 0 | 0 | 0 | 0 | 0 | 0 | 0 | 0 | 0 |
| mIGHV8-8 | 0.6 | 0.5 | 0.4 | 0.5 | 0.3 | 1.1 | 0.2 | 0.4 | 0.3 | 0.5 | 0.6 | 0.5 |
| mIGHV8S9 | 0 | 0 | 0 | 0 | 0 | 0 | 0 | 0 | 0 | 0 | 0 | 0 |
| mIGHV8-11 | 0 | 0 | 0 | 0 | 0 | 0 | 0 | 0 | 0 | 0 | 0 | 0 |
| mIGHV8-12 | 0.2 | 0.2 | 0.1 | 0.3 | 0.1 | 0.3 | 0.2 | 0.1 | 0.1 | 0.4 | 0.1 | 0.2 |
| mIGHV9-1 | 0 | 1 | 0.2 | 0.2 | 0.3 | 0.3 | 0.2 | 0.3 | 0.2 | 0 | 0 | 0 |
| mIGHV9-2-1 | 0 | 0 | 0 | 0 | 0 | 0 | 0 | 0 | 0 | 0 | 0 | 0 |
| mIGHV9-2 | 0.1 | 0.3 | 0.2 | 0.1 | 0.1 | 0.2 | 0.1 | 0.1 | 0 | 0.1 | 0 | 0 |
| mIGHV9-3-1 | 0 | 0 | 0 | 0 | 0 | 0 | 0 | 0 | 0 | 0 | 0 | 0 |
| mIGHV9-3 | 4.5 | 2.9 | 1.9 | 3.5 | 2.2 | 4 | 0.5 | 1.1 | 0.7 | 0.4 | 0.2 | 0.2 |
| mIGHV9-4 | 0.3 | 0.4 | 0.1 | 0.2 | 3.6 | 0.7 | 0.1 | 0.1 | 0.1 | 0 | 0 | 0.1 |
| mIGHV9S7 | 0 | 0 | 0 | 0 | 0 | 0 | 0 | 0 | 0 | 0 | 0 | 0 |
| mIGHV9S8 | 0 | 0 | 0 | 0 | 0 | 0 | 0 | 0 | 0 | 0 | 0 | 0 |
| mIGHV10-1 | 5.2 | 9.7 | 7.1 | 6.9 | 5.4 | 5.8 | 4.2 | 5.9 | 3 | 4.3 | 2.1 | 4.7 |
| mIGHV10-3 | 2.2 | 2.4 | 2 | 2.2 | 1.7 | 1.5 | 1.1 | 2.2 | 2.8 | 0.9 | 1 | 0.7 |
| mIGHV10S3 | 0 | 0 | 0 | 0 | 0 | 0 | 0 | 0 | 0 | 0 | 0 | 0 |
| mIGHV10S4 | 0 | 0 | 0 | 0 | 0 | 0 | 0 | 0 | 0 | 0 | 0 | 0 |
| mIGHV11-1 | 0 | 0 | 0 | 0 | 0 | 0 | 0 | 0 | 0 | 0 | 0.6 | 0 |
| mIGHV11-2 | 10.9 | 15 | 13.8 | 6.3 | 1.8 | 4.9 | 4.5 | 7.3 | 4.5 | 17.3 | 12.4 | 18.1 |
| mIGHV12-1-1 | 0 | 0 | 0 | 0 | 0 | 0 | 0 | 0 | 0 | 0 | 0 | 0 |
| mIGHV12-3 | 0.4 | 0.5 | 0.2 | 0.2 | 0.1 | 0.1 | 0 | 0 | 0.1 | 0.5 | 0.2 | 0 |
| mIGHV13-1 | 0 | 0 | 0 | 0 | 0 | 0 | 0 | 0 | 0 | 0 | 0 | 0 |
| mIGHV13-2 | 0.1 | 0 | 0 | 0.1 | 0.1 | 0.1 | 0 | 0.1 | 0.1 | 0.1 | 0 | 0 |
| mIGHV14-1 | 0 | 0.1 | 0.2 | 0 | 0.2 | 0 | 0.1 | 0 | 0.1 | 0 | 0 | 0 |
| mIGHV14-2 | 0 | 0 | 0 | 0.2 | 0.1 | 0.1 | 0 | 0 | 0 | 0.1 | 0 | 0 |
| mIGHV14-3 | 0.9 | 0.6 | 0.6 | 0.6 | 0.7 | 0.6 | 0.4 | 0.6 | 0.6 | 0.1 | 0.6 | 0 |
| mIGHV14-4 | 0.6 | 0.7 | 0.3 | 0.7 | 0.6 | 0.3 | 0.3 | 0.7 | 1.3 | 0.1 | 0.4 | 0.7 |
| mIGHV14S4 | 0 | 0 | 0 | 0 | 0 | 0 | 0 | 0 | 0 | 0 | 0 | 0 |
| mIGHV15-2 | 0.1 | 0 | 0 | 0 | 0 | 0 | 0 | 0 | 0 | 0 | 0 | 0 |
| mIGHV16-1 | 0 | 0 | 0 | 0 | 0 | 0 | 0 | 0 | 0 | 0 | 0 | 0 |
